# Hypoxia-induced Slug SUMOylation enhances lung cancer metastasis

**DOI:** 10.1186/s13046-018-0996-8

**Published:** 2019-01-06

**Authors:** Pei-Fang Hung, Tse-Ming Hong, Che-Chang Chang, Chung-Lieh Hung, Yuan-Ling Hsu, Yih-Leong Chang, Chen-Tu Wu, Gee-Chen Chang, Nei-Li Chan, Sung-Liang Yu, Pan-Chyr Yang, Szu-Hua Pan

**Affiliations:** 1 Institute of Biomedical Sciences, Academia Sinica, Taipei, 106 Taiwan; 2 Institute of Clinical Medicine, National Cheng Kung University, Tainan, 701 Taiwan; 3 The Ph.D. Program for Translational Medicine, Taipei Medical University, Taipei, 110 Taiwan; 4 Department of Medicine, Mackay Medical College, New Taipei City, 252 Taiwan; 5 Division of Cardiology, Department of Internal Medicine, Mackay Memorial Hospital, Taipei, 104 Taiwan; 6 Graduate Institute of Medical Genomics and Proteomics, College of Medicine, National Taiwan University, No. 2, Syu-Jhou Rd, Taipei, 100 Taiwan; 7 Department of Pathology and Graduate Institute of Pathology, College of Medicine, National Taiwan University, Taipei, 100 Taiwan; 8 Division of Chest Medicine, Department of Internal Medicine, Taichung Veterans General Hospital, Taichung, 407 Taiwan; 9 Graduate Institute of Biochemistry and Molecular Biology, College of Medicine, National Taiwan University, Taipei, 100 Taiwan; 10 Department of Clinical Laboratory Sciences and Medical Biotechnology, College of Medicine, National Taiwan University, Taipei, 100 Taiwan; 11 Department of Internal Medicine, College of Medicine, National Taiwan University, Taipei, 100 Taiwan; 12 Center of Genomic Medicine, National Taiwan University, Taipei, 100 Taiwan; 13 Ph.D. Program in Translational Medicine, National Taiwan University and Academia Sinica, Taipei, 100 Taiwan; 14 Genome and Systems Biology Degree Program, National Taiwan University and Academia Sinica, Taipei, 106 Taiwan

**Keywords:** Slug, SUMOylation, Hypoxia, Metastasis, Lung cancer

## Abstract

**Background:**

The Slug-E-cadherin axis plays a critical role in non-small-cell lung cancers (NSCLCs) where aberrant upregulation of Slug promotes cancer metastasis. Now, the post-translational modifications of Slug and their regulation mechanisms still remain unclear in lung cancer. Hence, exploring the protein linkage map of Slug is of great interest for investigating the scenario of how Slug protein is regulated in lung cancer metastasis.

**Methods:**

The Slug associated proteins, Ubc9 and SUMO-1, were identified using yeast two-hybrid screening; and in vitro SUMOylation assays combined with immunoprecipitation and immunoblotting were performed to explore the detail events and regulations of Slug SUMOylation. The functional effects of SUMOylation on Slug proteins were examined by EMSA, reporter assay, ChIP assay, RT-PCR, migration and invasion assays in vitro, tail vein metastatic analysis in vivo, and also evaluated the association with clinical outcome of NSCLC patients.

**Results:**

Slug protein could interact with Ubc9 and SUMO-1 and be SUMOylated in cells. Amino acids 130–212 and 33–129 of Slug are responsible for its binding to Ubc9 and protein inhibitor of activated STAT (PIAS)y, respectively. SUMOylation could enhance the transcriptional repression activity of Slug via recruiting more HDAC1, resulting in reduced expression of downstream Slug target genes and enhanced lung cancer metastasis. In addition, hypoxia could increase Slug SUMOylation through attenuating the interactions of Slug with SENP1 and SENP2. Finally, high expression Slug and Ubc9 levels were associated with poor overall survival among NSCLC patients.

**Conclusions:**

Ubc9/PIASy-mediated Slug SUMOylation and subsequent HDAC1 recruitment may play a crucial role in hypoxia-induced lung cancer progression, and these processes may serve as therapeutic targets for NSCLC.

**Electronic supplementary material:**

The online version of this article (10.1186/s13046-018-0996-8) contains supplementary material, which is available to authorized users.

## Background

Slug belongs to the Snail superfamily of transcriptional repressors, which contains five zinc finger domains near its *C*-terminus that recognize E-box elements, and a SNAG transrepressor domain at its *N*-terminus that is responsible for regulating the transcriptional repression activity [1]. Recent evidences suggest that Slug participates in epithelial-mesenchymal transition (EMT) during embryonic development and cancer metastasis by suppressing the expressions of its downstream target genes like E-cadherin, occludin, claudin-1, and integrin α3 [2–8]. Previously, we showed that p53 induced ubiquitin-mediated proteasome degradation of Slug via p53–Mdm2–Slug complex formation and mutation of p53 might induce Slug accumulation by repressing E-cadherin expression and resultant poor clinical outcomes in NSCLC [9]. All these findings suggest the crucial role of Slug modification in cancer progression.

In general, SUMOylation can facilitate protein stabilization, localization, and gene transcription [10]. Small ubiquitin-like modifier (SUMO) family, consists of SUMO-1, 2 and − 3, are initially expressed as inactive precursors, which mediates to expose a *C*-terminal double-glycine motif by SUMO-specific proteases [11, 12]. Then, activated SUMOs covalently attach to the ε-amino group of a lysine within the consensus sequence ψKXE via specific conjugation to a SUMO-activating enzyme heterodimer (E1), the unique SUMO-conjugating enzyme 9 (E2; Ubc9), and a SUMO protein ligase (E3) [13, 14]. Recent evidences indicated that non-consensus SUMO acceptor sites have also been found [15, 16]. Usually, E3 enzyme is not required for the addition of SUMO to target proteins in mammals, although the existence of specific E3 ligases can promote the conjugation of SUMO from E2 to its target protein [17]. Known SUMO E3 enzymes, PIASs and Pc2 participate additionally in gene transcription [18, 19]. Besides, SUMOylation is a dynamic process, where deSUMOylation is catalyzed by sentrin-specific proteases (SENPs) [20]. Recent reports show that SUMOylation plays important roles in tumorigenesis [21, 22]; however, the regulation mechanism remains as a mystery.

Recently, we utilized yeast two-hybrid system to identify potential Slug-interacting proteins; and found that components of the SUMOylation process were present in the pools isolated using Slug as prey. Herein, we detail dissect the regulation mechanism and the crucial role of SUMOylation in controlling Slug-mediated transcriptional repression; and also demonstrate that hypoxia could regulate Slug SUMOylation by reducing its deSUMOylation and result in cancer malignancy in NSCLC.

## Methods

### Cell line and culture conditions

The human embryonic kidney cell lines, HEK293 (ATCC® CRL-1573™) and HEK293T (ATCC® CRL-3216™) were purchased from American Type Culture Collection (ATCC, Manassas, VA). The human lung adenocarcinoma cell lines CL1–2 and CL1–5 are two sublines, selected from the CL1–0 cells via transwell invasion assays, with progressive invasiveness in a similar genotypic background [23]. The NCI-60 NSCLC cell line, Hop62, was a kind gift from Dr. Ker-Chau Li (Institute of Statistical Sciences, Academia Sinica). The cells were cultured in DMEM or RPMI 1640 medium supplemented with 10% fetal bovine serum (all from Invitrogen, Eugene, OR), 1% penicillin and streptomycin (all form Sigma−Aldrich, St. Louis, MO) in a humidified atmosphere containing 5% CO_2_ at 37 °C.

### Plasmid constructs

Human full-length Slug (GeneBank™ accession number NM_003068.4), SUMO-1 (NM_003352.4), SUMO-2 (NM_006937.3), SUMO-3 (NM_006936.2), PIAS1 (NM_016166.1), PIAS2 (NM_173206.2), PIAS3 (NM_006099.3), PIASy (NM_015897.2), and Pc2 (NM_003655.2) were PCR amplified and cloned into pBMT116, pCIneo-HA3, p3xFlag-CMV-7.1–2 and pFlag-CMV-2, pEGFP (Clontech, Mountain View, CA) and pcDNA3.1-HA vectors. Slug mutations were generated via PCR-directed mutagenesis according to the manufacturer’s instructions (QuickChange kit; Stratagene). The human active form and mutant construct of SUMO-1, Flag–Ubc9, wild-type and dominant negative mutant (C/S) SENP1/2 were kindly provided by Dr. Hsiu-Ming Shih (Institute of Biomedical Sciences, Academia Sinica), and the pcDNA3-HDAC1–Flag plasmid was kindly provided by Dr. Wen-Ming Yang (Institute of Molecular Biology, National Chung Hsing University).

### Yeast two-hybrid assay

Yeast two-hybrid screening was performed as described previously [24]. The DNA fragment encoding full-length human Slug was sub-cloned into the pBTM116 vector to produce LexA-Slug as bait. And it was used to screen against with human prostate cancer cDNA library. Approximately 10^5^ transformants were selected and PCR amplified to check as the potential interacting candidates. Then, LexA-Slug/ MST3 and Gal-Ubc9/ SUMO-1 constructs were co-transformed into L40 yeast. After overnight incubation, the resulting yeast cells were cultured in medium lacking tryptophan and leucine for selection of diploid cells. Diploid cells were further transferred to plate lacking tryptophan, leucine, and histidine and X-gal for 5 days to confirm the protein-protein interactions.

### In vitro pull-down assay

The GST–Ubc9 were expressed and purified as described previously [25]. In vitro transcription and translation were performed using the TNT SP6/T7 Quick Coupled Transcription/Translation System (Promega, San Luis Obispo, CA) according to the manufacturer’s instructions. GST fusion proteins expressed by bacteria were coupled to beads, and incubated with in vitro-translated proteins. After washing with buffer D (20 mM HEPES, pH 8.0, 20% v/v glycerol, 0.2 mM EDTA, 100 mM KCl, freshly added 0.5 mM PMSF and 0.5 mM DTT), the bound proteins were analyzed via electrophoresis on SDS–PAGE.

### Immunoprecipitation and immunoblotting assays

Immunoprecipitation and immunoblotting were performed as described previously [26]. The cells were lysed on ice for 5–10 min in RIPA lysis buffer (0.5% sodium deoxycholate, 0.1% sodium dodecyl sulfate, and 1% Nonidet P-40 in phosphate-buffered saline, all from Sigma) containing a 25-fold dilution of a stock protease inhibitor solution (Roche Diagnostics, Basel, Switzerland). The cell lysates were passed several times through a 21-gauge needle and clarified via centrifugation at 12,000 rpm for 30 min at 4 °C. The supernatants were taken as total cell lysates and precipitated with specific antibodies and protein A Sepharose. Then, the precipitated proteins were separated via SDS–PAGE and transferred to polyvinylidene difluoride (PVDF) membranes (Millipore, Billerica, MA) for immunoblotting with anti-Flag M2, anti-GFP, anti-β-actin (Sigma), anti-HA (Covance Research Products), anti-SUMO-1, anti-Ubc9, anti-Slug (A7, G18), anti-CtBP1, anti-Myc, anti-Lamin B, anti-Sin3A, anti-HDAC1, anti-HDAC2, anti-ubiquitin, anti-CD71 (Santa Cruz). anti-His (Qiagen, Hilden, Germany), anti-HIF1α, anti-GST (BD Transduction Laboratories), anti-acetylated lysine (Cell Signaling Technology), anti-HSP90 (Enzo Life Sciences, Farmingdale, New York) primary antibodies, followed with appropriate secondary antibodies conjugated with horseradish peroxidase and detected signals by Chemiluminescent Substrates (PerkinElmer).

### In vitro SUMOylation assays

The in vitro SUMOylation was performed in a 20-μl reaction mixture containing 2 mM ATP, 20 mM HEPES (pH 7.5), 5 mM MgCl_2_, 150 ng of an E1 SUMO ligase (Aos1/Uba2), 500 ng of Ubc9, 1 μg of SUMO-1 (all from LAE Biotechnology Co. Ltd., Taipei, Taiwan), and 2.5 μg of recombinant Nus–His–Slug protein at 30 °C for 2 h. For detecting SUMOylation in cells, the transfected cells were washed with PBS containing 20 mM *N*-ethylmaleimide (NEM, Sigma) and then lysed on ice with RIPA lysis buffer containing NEM and protease inhibitors. After incubation, the lysates were sonicated and centrifuged at 12,000 rpm at 4 °C for 30 min, and subsequently analyzed by immunoprecipitation and immunoblotting.

### Prediction of 3D protein structure

The amino acid sequences of human Slug (NP_003059.1) and PIASy (NP_056981.2) were obtained from the NCBI database. The protein structures were predicted by homology modeling using Discovery Studio 2.5 software.

### Reporter gene assays

The Slug binding region containing three tandem repeats of the Snail-binding site (SBS; 5’-AGC TTA GCA GGT GCA CGA TAT CAG CAG GTG CAC CAT ATG AGC AGG TGC AA-3′) and E-cadherin promoter sequences are described as previous [1, 27]. Cells cultured in 6-well plates were transfected using Lipofectamine 2000 according to the manufacturer’s protocol. Thirty-six hours after transfection, cell extracts were prepared using reporter lysis buffer, and luciferase activity was assessed using the dual luciferase reporter assay system (Promega Corp, Madison, WI) and a luminometer according to the manufacturer’s instructions. A control reporter expressing Renilla luciferase was used for normalization of the transfection efficiency.

### Electrophoretic mobility shift assays (EMSAs)

EMSAs were performed as previously described [28]. E-box C (5′-CGT CGG AAC TGC AAA GCA CCT GTG AGC TTG CGG AAG TC-3′) oligonucleotide probes were labeled with [γ-^32^P] ATP using T_4_ polynucleotide kinase. Binding reactions of in vitro-translated wild-type or mutant Slug protein and the labeled probes were performed according to the manufacturer’s instructions (Promega).

### Chromatin immunoprecipitation (ChIP)

The assays were performed using a Magna ChIP™ A/G Chromatin Immunoprecipitation Kit (Millipore) according to the manufacturer’s instructions. Briefly, equal numbers of cells were treated with 1% formaldehyde and then quenched with 0.125 M glycine for protein–DNA cross-linking. After washing with cold PBS, the cells were scraped, and soluble chromatin lysates were extracted via sonication and centrifugation. Two percent of the diluted chromatin solution was reserved as the total input sample. The diluted chromatin solution was incubated with anti-HDAC1 and normal mouse IgG antibodies overnight at 4 °C with rotation. Then, the DNA/protein solution was eluted with proteinase K containing elution buffer at 65 °C for 2 h to break the formaldehyde cross-links. DNA solution was used as the template for 33 cycles of PCR amplification using E-cadherin gene-specific primers.

### Reverse transcription-polymerase chain reaction (RT-PCR)

RNA was isolated from cells using TRIzol reagent (Invitrogen) according to the manufacturer’s instructions. Total RNA was reverse transcribed for 60 min at 50 °C using Super Script III Reverse Transcriptase (Invitrogen) and Random Hexamer primers (Applied Biosystems, Foster City, CA) in the presence of an RNase inhibitor. And the gene expressions were analyzed by PCR using the following primer sequences: Slug forward, 5′-CAT GCC ATT GAA GCT GAA AAG-3′, and reverse, 5′-GCA GTG AGG GCA AGA AAA AG-3′; Gβ-like forward, 5-GTA TGG AAC CTG GCT AAC TG-3′, and reverse, 5′-TAC TGA TAA CTT CTT GCT TC-3′; E-cadherin forward, 5′-GCT GGA GAT TAA TCC GGA CA-3′, and reverse, 5′-ACC CAC CTC TAA GGC CAT CT-3′; claudin 1 forward, 5′-CCG TTG GCA TGA AGT GTA TG-3′, and reverse, 5′-GTT TTG GAT AGG GCC TTG GT-3′; occludin forward, 5′-GAT GAG CTG GAG GAG GAC TG-3′, and reverse, 5′-GCT CAC AGA GGT TTG GCT TC-3′; and integrin α3 forward, 5′-GCC TGC CAA GCT AAT GAG AC-3′, and reverse, 5′-ATC TCC GTG GGA TAC AGC AG-3′.

### Modified Boyden chamber invasion assay

Modified Boyden chambers with polycarbonate-membrane inserts (pore size, 8 μm; Falcon; Becton Dickinson, Franklin Lakes, NJ) coated with 12 μg of Matrigel (BD; San Jose, CA) were used for cell invasion assays. Stable transfectants were suspended in culture medium containing 10% NuSerum (Invitrogen). Cells (2.5 × 10^4^) were placed in the upper chambers, and 1 ml of medium was placed in the lower chambers. After incubation for 24 h at 37 °C, the cells were fixed with methanol for 10 min at room temperature and then stained for 30 mins at room temperature with a 50 μg/ml solution of propidium iodide (PI) (Sigma). The number of cells on each membrane was counted under a microscope at a magnification of 50× using the Analytical Imaging Station software package (Imaging Research Inc., St. Catharines, ON, Canada).

### Experimental metastasis assay in vivo

A single-cell suspension containing 10^6^ cells in 0.1 ml of PBS was injected into the lateral tail vein of 6-week-old SCID mice (*n* = 9 per group). Seventy-nine days after injection, the mice were sacrificed under carbon dioxide anesthesia, and their lungs were removed and fixed in 10% formalin. The lung tumor cell nodules were counted under a dissecting microscope. Embedded tissues were sliced into 4-μm-thick sections, and the sections were stained with hematoxylin and eosin for histological analysis. All mouse experiments were performed and approved by the Laboratory Animal Center, National Taiwan University College of Medicine.

### Patient specimens

Lung tumor tissue specimens were obtained from 104 and 85 patients who underwent surgical resection for histologically confirmed NSCLC at the Taichung Veterans General Hospital (TVGH, Taichung, Taiwan) from 2000 to 2004 and National Taiwan University Hospital (NTUH, Taipei, Taiwan) from 1996 to 2005, respectively. None of the patients had received preoperative adjuvant chemotherapy or radiation therapy; and the post-surgical pathologic stage of each tumor was classified according to the international TNM classification system [29]. All the investigations were approved by the Institutional Review Boards of TVGH and NTUH.

### Real-time quantitative polymerase chain reaction (Q-PCR)

The Slug and Ubc9 transcript levels were determined via Q-PCR using an ABI Prism 7900 sequence detection system (Applied Biosystems) according to the manufacturer’s instructions. The designed primers and probe sets for Slug (Hs00161904_m1) and Ubc9 (Hs00163336_m1) were purchased from Applied Biosystems; and DEAD box helicase 5 (DDX5; Hs00189323_m1) was used as the internal control. The cut-off value of 0.6 was used to divide patients into high- and low-expression groups.

### Immunohistochemistry

Four-μm-thick paraffin-embedded tumor tissue sections were deparaffinized in Trilogy Solution (Cell Marque Corp., Rocklin, CA) at 121 °C for 10 min. Samples were then treated with 3% H_2_O_2_-methanol and subsequently incubated with DakoCytomation Dual Endogenous Enzyme Block (DakoCytomation Inc., Carpinteria, CA) for 10 min, Ultra V Block (LAB VISION Corporation, Fremont, CA) for 10 min, antibody dilution buffer (Ventana Medical Systems Inc., Tucson, Arizona) for 10 min, and the Slug (Abgent, San Diego, CA) or Ubc9 antibodies overnight at 4 °C. Immunoreactivity was detected using the BioGenex Super Sensitive Link-Label IHC Detection System (BioGenex, San Ramon, CA) according to the manufacturer’s instructions.

### Statistical analysis

The quantitative in vitro and in vivo data were analyzed using Student’s *t*-tests. All statistical tests were 2-sided, and *P* values of less than 0.05 were considered statistically significant. In addition, the curves obtained from the cell migration assay and from analyses of clinical patient samples were examined via one-way ANOVA and the log rank test.

## Results

### Identification of Ubc9 and SUMO-1 as Slug-interacting proteins

**Fig. 1:**
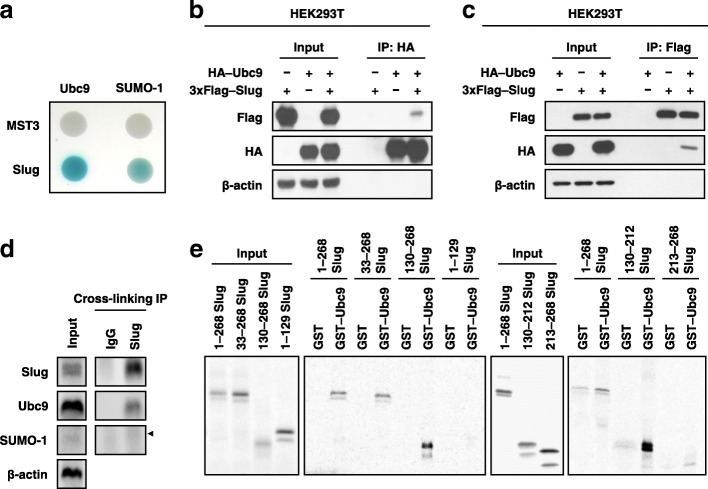
Slug interacts with components of the SUMOylation pathway. **a** Slug was found to interact with Ubc9 and SUMO-1 based on a yeast two-hybrid screen. MST3 was used as a negative control. **b-d** The association of Slug with Ubc9. Lysates of HEK293T cells transfected with the indicated plasmid (**b** and **c**) were used to perform exogenous immunoprecipitation assays. **d** CL1–5 cell lysate were treated with 1% formaldehyde to performed endogenous immunoprecipitation assays. Cross-linked Slug complexes were analyzed using the indicated antibodies for immunoblotting. β-actin was used as the internal control. **e** Direct interaction of Slug with the Ubc9 protein. Recombinant proteins, as indicated, were examined via a pull-down assy

As Slug is a key EMT regulator [30], we used yeast two-hybrid system to identify specific Slug-interacting proteins and to elucidate how Slug functions in EMT progression. The results indicated that Ubc9 and SUMO-1, two SUMOylation pathway members, were present in the pools. Next, the interactions of Ubc9 and SUMO1 with Slug were confirmed by β-galactosidase activity on solid medium; serine/threonine-protein kinase 24 (MST3) was used as the negative control (Fig. 1a). Then, Slug-Ubc9 interaction was further examined by exogenous immunoprecipitation assay in HEK293T cells (Fig. 1b and c) and endogenous immunoprecipitation assay with cross-linking for Slug protein in CL1–5 cells (Fig. 1d). Therefore, we finally identified that Ubc9 and SUMO-1 are two Slug-interacting partners in human lung cancer cells.

### Amino acids 130–212 of Slug are crucial for its interaction with Ubc9

To determine the region of Slug that interacts with Ubc9, we generated several deletion mutants of Slug for glutathione *S*-transferase (GST) pull-down assays. Slug fragments consisting of amino acids 1–268 (full-length), 33–268, 130–268, or 130–212, but not fragments 1–129 and 213–268, were pulled down by GST–Ubc9 (Fig. 1e). Thus, amino acids 130–212 of Slug are crucial for interacting with Ubc9.

**Fig. 2:**
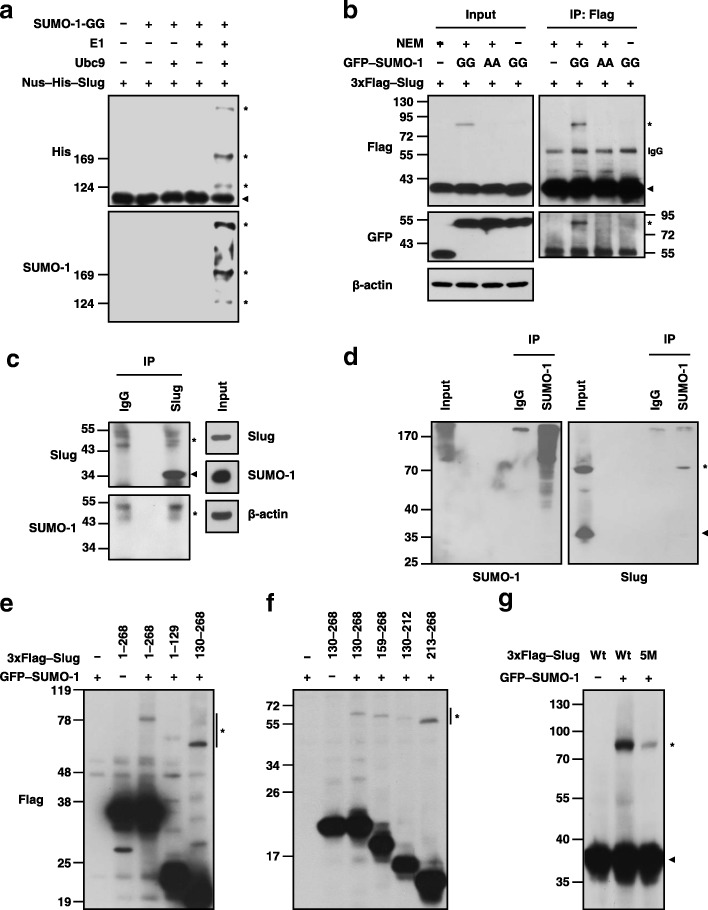
Slug is covalently modified by SUMO-1. **a** Identification of Slug SUMOylation using the in vitro SUMOylation assay system. The purified proteins were incubated and then analyzed by immunoblotting using the indicated antibodies. **b** Exogenous Slug SUMOylation. The lysates of HEK293T cells transfected with the indicated plasmids and treated or not treated with NEM were collected and subjected to immunoprecipitation using an anti-Flag antibody. The results were analyzed by immunoblotting with the indicated antibodies. **c, d** Endogenous Slug SUMOylation*.* The lysates of CL1–5 cells treated with NEM were immunoprecipitated with an anti-Slug (**c**) or anti-SUMO-1 antibody (**d**); the precipitates were individually resolved in reducing (**c**) or non-reducing (**d**) sample buffer. Immunoblotting was performed using anti-Slug and anti-SUMO-1 antibodies to examine the endogenous SUMOylation of Slug. **e-g** Lysates of HEK293T cells cotransfected with different fragments or mutant forms of Slug were harvested and then subjected to immunoblotting with an anti-Flag antibody. The results were analyzed by immunoblotting with the indicated antibodies. The asterisk and arrowhead indicate Slug modified and not modified with SUMO-1, respectively

SUMO-1 modifies slug in vitro Because Slug interacted with both Ubc9 and SUMO-1, we explored whether Slug could be SUMOylated by SUMO-1. An in vitro SUMOylation assay was performed using purified Slug proteins as the substrate together with various combinations of E1, E2, and a mature form SUMO-1 (SUMO-1-GG). Immunoblotting revealed a shifted band only in the presence of E1, E2, and SUMO-1 (Fig. 2a). Subsequently, we examined whether Slug protein could be SUMOylated in cells; in this experiment, HEK293T cells were transfected with 3xFlag-tagged Slug and GFP-tagged SUMO-1 vectors. High-molecular-weight SUMOylated Slug was detected in cells co-expressing Slug and SUMO-1 but not in cells expressing Slug alone (Fig. 2b). To validate that the shifted band was SUMO-1 conjugated Slug, we utilized SUMO-1-AA, which cannot bind to substrates because it lacks a *C*-terminal double-glycine motif, and the SUMO isopeptidase inhibitor *N-*ethylmaleimide (NEM) to determine this event. The shifted band observed in samples of SUMO-1-GG and Slug was more obvious than that of SUMO-1-AA and Slug or SUMO-1-GG and Slug without NEM treatment (Fig. 2b). Moreover, endogenous Slug SUMOylation was also detected in CL1–5 cells (Fig. 2c, d). Therefore, Slug is conjugated with SUMO-1 in cells*.*

As SUMO1–3 are three key members in SUMO family, whether SUMO-2 and SUMO-3 could like SUMO-1 to modify Slug protein should be further clarified. To address this issue, we transfected different SUMO isoform plasmids into cells and found that all SUMO isoforms can modify Slug with SUMO-1 showed predominantly Slug SUMOylation rather than SUMO-2 and SUMO-3 (Additional file 1: Figure S1).

### Slug SUMOylation primarily occurs within its 213–268 amino acid region

To explore the target regions of Slug responsible for its SUMOylation, we generated several Slug truncated mutants and examined their SUMOylation patterns in cells. HEK293T cells were cotransfected with different Slug truncated mutants and the GFP–SUMO-1 plasmids. The data showed that the shifted band was detected in cells expressing Slug fragments 1–268 and 130–268 (Fig. 2e); this result implied that SUMOylation primarily occurs in Slug *C*-terminal region. Further dissection the *C*-terminus of Slug identified that fragments 213–268 exhibited high SUMOylation ability (Fig. 2f), suggesting the critical region for Slug SUMOylation.

Sequence analysis indicated that five lysine residues located within Slug fragment 213–268 (K239, K240, K244, K248, and K258), especially amino acids 257–260 (HKHE), matched well with the SUMOylation consensus sequence (ψKXE). Therefore, we established Slug5M, in which lysine 239, 240, 244, 248, and 258 residues were substituted with arginine, and determine its SUMOylation level. The results showed the intensity of the shifted band for Slug5M was decreased approximately 75% than that for the wild-type Slug (Slug5M/wild-type Slug = 27.48 ± 13.87%, Fig. 2g). In order to find out all the SUMOylated sites of Slug, we further mutated all lysine residues (Slug22M) and individually substituted them with lysine residue. The results showed that in addition to the 5 sites we identified, lysine 188 was also important for Slug SUMOylation (Additional file 2: Figure S2a). As such, we further created two Slug mutants, Slug6M (K188, 239, 240, 244, 248, and 258R) and Slug8M (K188, 192, 211, 239, 240, 244, 248, and 258R), to examine their effects on Slug SUMOylation levels, repression activities and their DNA binding abilities. Like our expected, the SUMOylation levels and transcriptional repression activities of Slug were significantly decreased with the increasing number of lysine mutations on Slug protein (Additional file 2: Figure S2b and 2c); but the DNA binding abilities of Slug6M and Slug8M were also simultaneously attenuated (Additional file 2: Figure S2d). As such, Slug5M is more suitable than the other two mutants for studying the functional events of Slug SUMOylation.

### The E2 Ubc9 can promote Slug SUMOylation

**Fig. 3:**
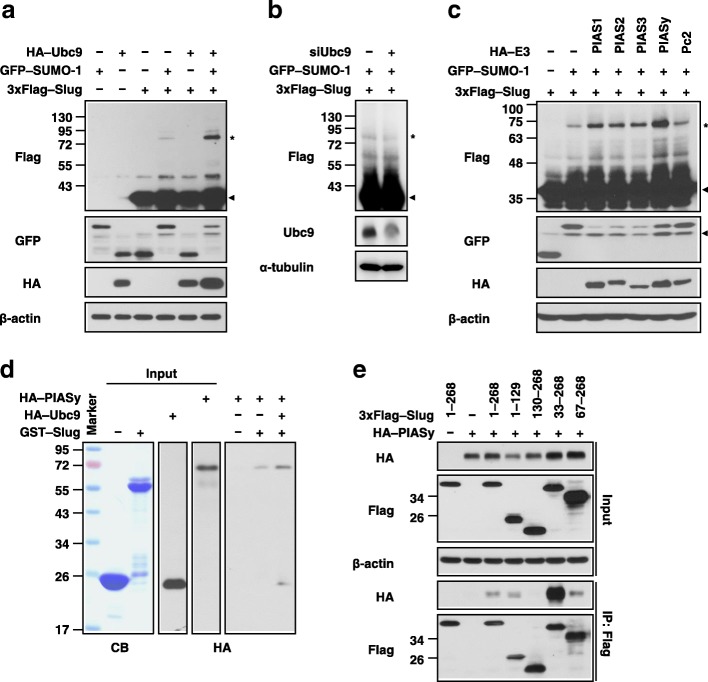
The regulation and components of Slug SUMOylation. **a, b** Ubc9 influences Slug SUMOylation. Lysates of HEK293T cells transfected with the indicated plasmids or siRNA were analyzed by immunoblotting with the indicated antibodies. **c** E3 enzymes involved in Slug SUMOylation. Lysates of HEK293T cells transfected with different E3 constructs were analyzed by immunoblotting with the indicated antibodies. β-actin was used as the internal control. **d** Direct interaction of Slug with PIASy. The recombinant GST and GST–Slug proteins produced from bacteria were shown by Coomassie blue (CB) staining. The products of the in vitro transcription/translation system and the results of the pull-down assay were analyzed by immunoblotting using anti-HA antibodies. **e** Domain mapping of the interaction between Slug and PIASy. HEK293T cells were cotransfected with Slug fragments of varying lengths. The lysates were examined by immunoprecipitation with anti-Flag antibodies. The results of immunoblotting with the indicated antibodies are shown

Since Ubc9 is the unique E2 for SUMOylation and upregulated in cancers [31], we examined whether Slug SUMOylation levels could be interfered through manipulating Ubc9 expressions in vitro. As Fig. 3a showed, the intensity of the shifted band became higher when we overexpressed Ubc9 in cells. Conversely, the intensity became lower when we silenced Ubc9 in cells (Fig. 3b). Moreover, stable Slug-overexpressing cells infected with virus of control vector or Ubc9 were subcutaneously injected into the mice and the resulting tumors were harvested for examining this event in vivo after 6 weeks by immunoblotting. The results showed that the SUMOylated level of Slug was higher in tumors from Slug- and Ubc9-overexpressing cells than those from the vector control (Additional file 3: Figure S3). All these suggest the existence of Ubc9 could enhance Slug SUMOylation both in vitro and in vivo; and the expressions of Slug and Ubc9 could be used as an indicator of SUMOylated Slug level.

### The *N*-terminal region of Slug directly interacts with the E3 PIASy

The PIAS family proteins or Pc2 are well known E3 ligase for SUMOylation [32, 33]; this let us further investigated whether these proteins play a central role in Slug SUMOylation. The data indicated that Slug SUMOylation was facilitated by PIAS1, PIAS2, PIAS3, and PIASy but not by Pc2 (Fig. 3c).

As literature revealed that E3 proteins can facilitate SUMOylation via direct or indirect interactions [34], we were interested in the relationship between Slug and PIASs. Using a GST pull-down assay, we examined the association between Slug and PIASs. The results showed that all PIASs, especially PIASy, directly interacted with GST–Slug rather than GST alone (Additional file 4: Figure S4). Then, we used PIASy as a material to further clarify the detail process of Slug SUMOylation. First, we tested whether the presence of Ubc9 could affect the Slug-PIASy interaction. The results indicated that the amount of pulled-down PIASy was greater in the presence of Ubc9 than in the absence of Ubc9 (Fig. 3d), indicating that Slug can form a complex with both Ubc9 and PIASy. Next, the specific binding domain of Slug to PIASy was determined via co-immunoprecipitation assays using HEK293T extracts from cells co-expressing 3xFlag–Slug truncated mutants and HA–PIASy. Our findings showed that PIASy interacted with Slug fragments 1–268, 1–129, 33–268, and 67–268 but not with the Slug *C*-terminus (fragments 130–268) (Fig. 3e), suggesting that the Slug *N-*terminus may be responsible for its interaction with PIASy.

### Proposed three-dimensional (3D) interaction model of Slug, Ubc9, and PIASy

According to the results of Slug domain mapping, we found that the binding regions of Ubc9, PIASy and SUMO-1 were distinct in the linear structure (Additional file 5: Figure S5a). This raises the issue how the presence of Ubc9 enhanced the Slug-PIASy interaction (Fig. 3d) and mediated Slug SUMOylation. Recent studies indicated that the molecules E3, Ubc9, SUMO and their substrates might bring into close proximity, form complexes and subsequently facilitate SUMOylation [34, 35]. This let us try to produce a 3D structural model combining previously published data (Ubc9 and SUMO-1) and a chimeric structure (Slug and PIASy) to understand the stereochemistry of these Slug SUMOylation related components (Additional file 5: Figure S5b). The producing model supports our hypothesis that Slug can form a complex with Ubc9 and PIASy, and the closer three-dimensional space may help the occurrence of Slug SUMOylation.

### SUMOylated Slug is present on chromatin

**Fig. 4:**
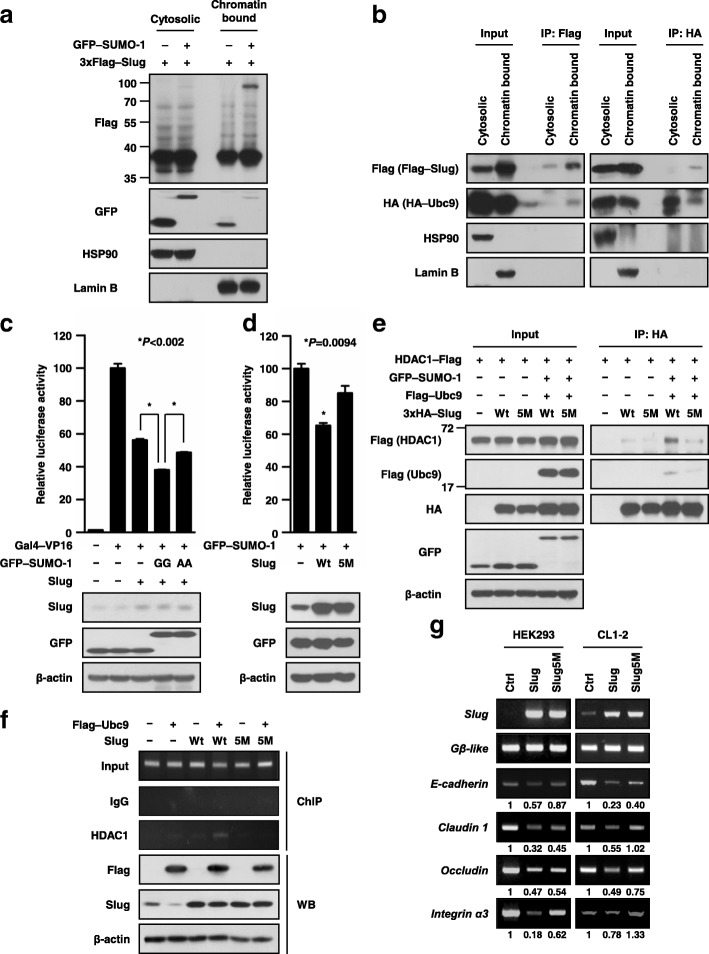
SUMOylation enhances the transcriptional repression activity of Slug. **a** Distribution of SUMOylated Slug in the cytosolic and chromatin-bound fractions. Cytosolic HSP90 protein and chromatin-bound Lamin B were used as controls for cellular fractionation. **b** The interaction of Slug with Ubc9 in the cytosolic and chromatin-bound fractions. HEK293T cells co-expressing 3xFlag-labelled Slug and HA–Ubc9 were harvested and immunoprecipitated using anti-Flag antibodies. HSP90 and Lamin B are shown as fractionation controls. **c, d** SUMOylation enhances the transcriptional repression activity of Slug. HEK293T cells were cotransfected with the indicated plasmids and a reporter vector driven by SBS–Gal4 (**c**) or by the E-cadherin promoter (**d**). Luciferase activities and immunoblotting were evaluated 36 h after transfection. The activity induced by Gal4–VP16 alone (**c**) or basal activity (**d**) was normalized to 100%. All data were reported as mean values ± SEM, and *P* values were calculated via Student’s t-test. The asterisk represents a *P* value of < 0.05 compared to the group stimulated with Gal4–VP16 alone or the basal activity level. **e** SUMOylation increases the recruitment of HDAC1. Lysates of HEK293T cells cotransfected with the indicated plasmids were subjected to immunoprecipitation using an anti-HA antibody. **f** Top: The indicated antibodies were used to pull down protein-DNA complexes, and the E-cadherin promoter level in the samples was determined by PCR using a gene-specific primer set. Input, an aliquot of each sample was prepared and used as a template for PCR to examine the level of the E-cadherin promoter before immunoprecipitation (IP). Bottom: Western blot analysis of the indicated proteins was performed on the products of ChIP. **g** SUMOylation affects the expression of Slug-regulated downstream targets. HEK293 and CL1–2 cells were induced to express the wild-type and mutant Slug, respectively, using a lentiviral system. The mRNA expression of the indicated genes was determined via RT-PCR. Gβ-like was used as the internal control

Furthermore, we were curious about where Slug SUMOylation may occur in cell. To solve this, cells were first separated into cytosolic and chromatin-bound fractions to identify the existence of SUMOylated Slug. The results showed that most SUMOylated Slug was presented in the chromatin-bound fraction but rarely found in the cytosolic fraction (Fig. 4a). Then, we determined the compartments in which Slug and Ubc9 interact via performing co-immunoprecipitation on each cellular fraction. The data showed that the DNA-bound form of Slug interacts with Ubc9 leading to Slug SUMOylation (Fig. 4b).

### Slug SUMOylation enhances its transcriptional repression activity

Since Slug is a well-known transcriptional repressor, whether SUMOylation can affect its repression activity should be further clarified. As such, we analyzed the repression activities of Slug and Slug5M by Snail-binding site (SBS)–Gal4 promoter and E-cadherin promoter, respectively. First, HEK293T cells were co-transfected with the SBS–Gal4–luciferase reporter and Gal4–VP16 activator expression plasmids together with the Slug plasmid and various SUMO-1 mutants. As previous report, Slug could repress the expression of the reporter gene [1]. We showed the repression activity of Slug was greater in cells co-expressing Slug and SUMO-1-GG than in cells expressing Slug alone or co-expressing Slug and SUMO-1-AA (Fig. 4c). Similar results were re-confirmed by E-cadherin promoter region, a well know downstream target of Slug [3], that wild-type Slug suppressed E-cadherin promoter expression greater than Slug5M (Fig. 4d); and the immunoblotting data demonstrated the expression of the indicated proteins (Fig. 4c, d).

As the DNA-binding ability of Slug does not change with Slug5M (Additional file 6: Figure S6a), we then examined whether the decreasing transcriptional suppression activities of Slug5M is due to its’ protein instabilities. Through cycloheximide treatment, we found that the protein stabilities of wild-type Slug and Slug5M were not significantly different (Additional file 6: Figure S6b). Taken together, these findings suggest that Slug SUMOylation may enhance its transcriptional suppression activity.

### Slug SUMOylation increases the recruitment of HDAC1

In the previous, HDAC1/2 corepressor complex had been identified could interact with Slug [36]. Therefore, we verified whether SUMOylation could affect this complex formation. As Fig. 4e showed that HDAC1 were associated with both wild-type Slug and Slug5M, but only wild-type Slug overexpressed with Ubc9 and SUMO-1 displayed increasing recruitment of HDAC1. Moreover, wild-type Slug interacted with Sin3A, HDAC1, HDAC2 and CtBP1 more abundantly than Slug5M in the nucleus (Additional file 7: Figure S7).

To investigate whether the increasing Slug-HDAC1 complex can enhance the suppression activity of Slug, the occupation status of endogenous HDAC1 on the E-cadherin promoter was first examined by chromatin immunoprecipitation (ChIP) assays. As expected, HDAC1 more strongly associated with the E-cadherin promoter in cells overexpressing wild-type Slug and Ubc9 than in Slug5M and Ubc9 (Fig. 4f); and this result was also re-confirmed in human lung adenocarcinoma cell line, Hop62 (Additional file 8: Figure S8). Furthermore, we analyzed the RNA expressions of several downstream targets in cells infected with the empty vector (Ctrl), wild-type Slug, or Slug5M via reverse transcriptase polymerase chain reaction (RT-PCR). The data showed that the expressions of all target genes were suppressed when wild-type Slug was expressed in HEK293, CL1–2 and CL1–5 cells, and their expressions was partially restored when Slug5M was expressed (Fig. 4g and Additional file 9: Figure S9). Collectively, all these findings implied that Slug SUMOylation might increase HDAC1 recruited to the promoter region of its downstream target genes and enhance the repression activity of Slug.

### Hypoxia induces the accumulation of SUMO-1 on Slug by decreasing deSUMOylation

**Fig. 5:**
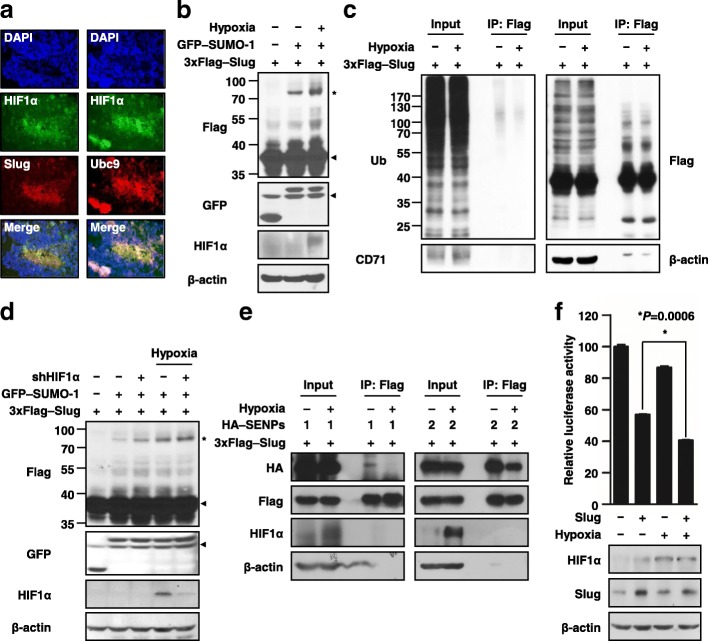
Hypoxia increases Slug SUMOylation. **a** IHC analysis of xenograft tumor section. Adjacent serial sections were stained for Slug and Ubc9 combined with HIF1α. The signal was captured by a fluorescent microscope (original magnification, × 200). **b** Hypoxia increased the SUMOylation of Slug. Cells were exposed to normoxia or hypoxia (1% O_2_) for 4 h before harvesting. Whole cell lysates were analyzed by immunoblotting with the indicated antibodies. **c** The ubiquitination level of Slug under hypoxic conditions. HEK293T cells were transfected with 3xFlag-tagged Slug. After 36 h of transfection, the cells were treated with MG132 under normoxic or hypoxic conditions. Immunoprecipitation was performed using an anti-Flag antibody prior to immunoblotting with the indicated antibodies. The accumulation of CD71 was used to confirm that cells were exposed to hypoxia. **d** Hypoxia promoted the transcriptional repression activity of Slug. After transient transfection with the indicated gene constructs and a reporter vector driven by the E-cadherin promoter, the cells were incubated for 36 h. Before harvesting, the cells were exposed to normoxia or hypoxia and then subjected to luciferase assays. The relative luciferase activity levels were normalized to the levels of pGL4.74-TK. The data were reported as mean values ± SEM, and *P* values were calculated via Student’s t-test. The asterisk represents a *P* = 0.0006. **e** Slug SUMOylation did not occur via HIF1α protein activity. HEK293T cells were transfected with the indicated plasmid and then exposed to normoxia or hypoxia, followed by cell harvesting. The results of immunoblotting with the indicated antibodies are shown. The asterisk and arrowhead indicate Slug modified and not modified with SUMO-1, respectively. **f** A decrease in the Slug–SENP1/2 interaction in response to hypoxia. HEK293T cells were transiently transfected with the indicated plasmids and then exposed to hypoxia. Immunoprecipitation was performed using an anti-Flag antibody prior to immunoblotting with the indicated antibodies

Hypoxia, a characteristic of advanced solid tumors, could upregulate Slug or Ubc9 expression and could also increase global protein modification by SUMO-1 [37–40]. These let us wonder whether hypoxia could enhance the event of Slug SUMOylation. First, we assessed the expressions of Slug and Ubc9 in xenograft tumor specimen; and also examined the expressions of transcription factor, hypoxia-inducible factor 1-alpha (HIF1α), to evaluate their hypoxic status. The results showed that whether Slug or Ubc9 proteins were co-localized with HIF1α (Fig. 5a) suggesting that Slug SUMOylation may occur in the hypoxic region in tumor. Then, Slug SUMOylation levels were detected under hypoxia and normoxia in vitro. Our data indicated that the intensity of the shifted band was stronger in cells exposed to hypoxia than that of normoxia (Fig. 5b); though ubiquitination showed no significant difference between the normoxic and hypoxic groups (Fig. 5c). Moreover, the luciferase reporter assay also indicated that hypoxia could induce higher degree of Slug downstream reporter gene suppression than normoxia (Fig. 5d). Collectively, all these suggested that hypoxia could regulate Slug SUMOylation in vitro and in vivo.

Surprisingly, manipulating HIF1α expression did not affect Slug SUMOylation (Fig. 5e); and the interactions of Slug with Ubc9 and PIASy were only slightly increased under hypoxia (Additional file 10: Figure S10a and b). Since SUMO modification is a dynamic process, this let us further speculate whether hypoxia-induced Slug SUMOylation is through interfering the process of Slug deSUMOylation. As literature pointed that SENP1 and SENP2 proteins participate in SUMO-1 de-conjugation [20], we first examined which component was involved in Slug deSUMOylation. Using the expression of catalytically dead mutant (C/S) of HA–SENP1 or HA–SENP2, we found that both wild-type SENP1 and SENP2 rather than their C/S mutants could suppress Slug SUMOylation (Additional file 10: Figure S10c). Then, we detected the hypoxic effects on these interactions. Interestingly, we found that the Slug-precipitated SENP1 and SENP2 levels were dramatically reduced under hypoxia (Fig. 5f). These implied that hypoxia may enhance Slug SUMOylation through down regulation of the deSUMOylation rate in cells.

### Slug SUMOylation promotes tumor migration, invasion and metastasis

**Fig. 6:**
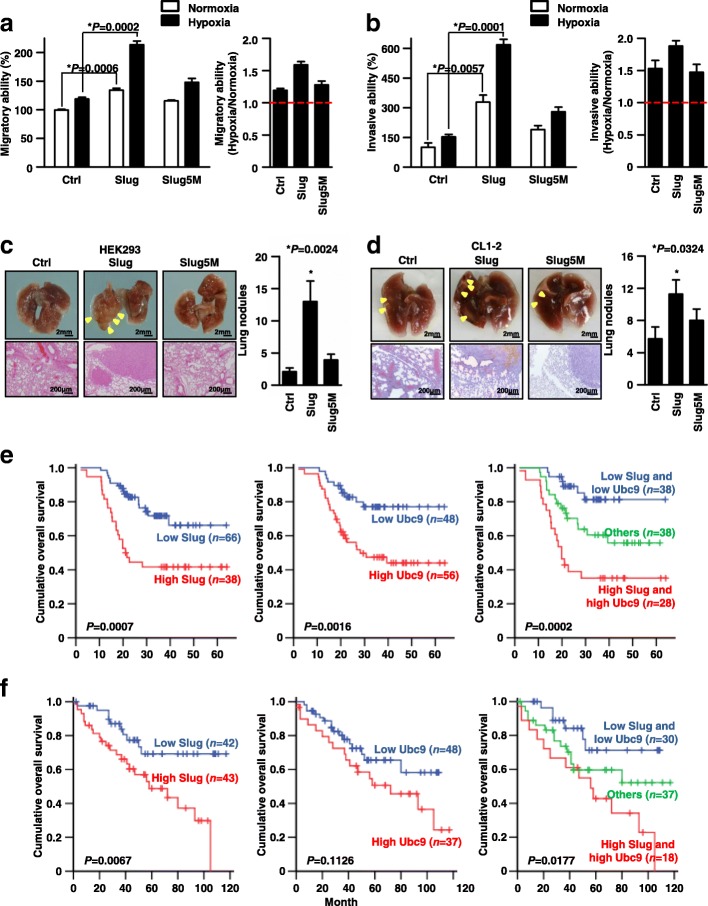
Cell migration, invasion and metastasis can be regulated by Slug SUMOylation. **a** Effect of hypoxia on the migration of cells expressing wild-type Slug or Slug5M. Successfully lentiviral infected HEK293/ Ctrl, Slug and Slug5M cells were cultured for 24 h and treated with hypoxia for an additional 4 h. Phase contrast images were captured at the beginning (0 h) and after 8 h of the cell migration assay using standard culture inserts. The migratory ability of the cells is presented in the Left panel, and the control group exposed to normoxia was normalized to 100%. **b** Cell invasion was assessed using the Matrigel-coated transwell system. The invasive cells were calculated and normalized to the results for control cells exposed to normoxia. The values are presented as the means ± SEM (*n* = 3). The right panel shows the fold-change in the number of invasive cells for each cell line under hypoxia compared to normoxia. **c**, **d** Mice were injected into the tail vein with HEK293 or CL1–2 cells overexpressing the control (Ctrl) or the wild-type (Slug) or mutant form (Slug5M). The number of lung metastatic nodules (arrowhead) was measured in 9 (**c**) or 7 (**d**) mice per group. The nature of the nodules was confirmed by hematoxylin and eosin staining. The error bars indicate the mean values ± SEM, and *P* values were calculated using Student’s t-test. The asterisk represents the *P* value of < 0.05 for the comparison of the labeled group to the control group. Scale bar: 2 mm in lung photo and 200 μm in HE staining. **e**, **f** Kaplan-Meier estimates of overall survival in NSCLC patients. The mRNA (**e**) and protein (**f**) levels of Slug and Ubc9 were determined via Q-PCR and immunohistochemistry, respectively (left and middle graphs, respectively). The patients were divided into the high- (red line) and low-expression (blue line) groups. The graph on the right shows the combined effect of Slug and Ubc9 on the overall survival of NSCLC patients. *P* values were calculated using the log rank test

Further to understand the impact of SUMOylation on Slug functions; the effects of Slug SUMOylation on cell migration and invasion should be determined. The results showed that the cells presented none significant difference in cell growth (Additional file 11: Figure S11), but the migratory ability of HEK293/Slug or Slug5M were greater than that of HEK293/Ctrl under normoxia; only the wild-type Slug reached significant (*P* = 0.0006). Further, the migratory effects of Slug wild-type and Slug5M were re-confirmed by overexpressing the vector control, Slug and Slug5M into two additional human lung cancer cell lines, CL1–2 and Hop62 under normoxia (Additional file 12: Figure S12a and b). Moreover, we also found that hypoxia could significantly enhance the difference between wild-type Slug- and Slug5M-increased cell migratory abilities (Fig. 6a, Left panel); and the combined effect of Slug and hypoxia were calculated (Fig. 6a, Right panel).

Additionally, overexpression of wild-type Slug and Slug5M in HEK293 cells could increase cell invasiveness by 3.3-fold and 1.9-fold relative to the vector control under normoxia, respectively (both *P* < 0.05; Fig. 6b, Left panel); an observed combined effect of wild-type Slug and hypoxia were also existed (Fig. 6b, Right panel). Similarity, the effects of Slug wild-type and Slug 5 M on cell invasiveness under normoxia were also observed in both CL1–2 and Hop62 lung cancer cells (Additional file 13: Figure S13a and b). Then, we extended the assessment on metastasis in vivo; cells were intravenously injected into the lateral tail vein of mice and the formation of pulmonary nodules was observed. HEK293/Slug developed more pulmonary nodules than HEK293/Ctrl and HEK293/Slug5M (mean number of pulmonary nodules: 2.1 ± 0.57 for HEK293/Ctrl, 13.0 ± 3.18 for HEK293/Slug, and 3.9 ± 0.91 for HEK293/Slug5M; HEK293/Ctrl vs. HEK293/Slug, *P* = 0.0024; Fig. 6c) with morphology of metastatic lung nodules displayed; and similar results were also seen by mice tail vein injected with CL1–2 lung cancer cells (CL1–2/Ctrl vs. CL1–2/Slug, *P* = 0.0324; Fig. 6d). Collectively, all these data indicated that SUMOylation may promote Slug-induced cancer metastasis in vitro and in vivo.

### Overexpression of Slug and Ubc9 associates with poor overall survival among NSCLC patients

Although our results consistently suggested that Slug SUMOylation could promote cell metastatic abilities both in vitro and in vivo, such studies do not fully reflect clinical malignancy. Accordingly, we examined the mRNA expressions of Slug and Ubc9 in primary tumor specimens from 104 NSCLC patients, with baseline characteristics showed in Table 1, by real-time quantitative PCR (Q-PCR). Similar to previous findings [31, 41], patients exhibiting higher Slug or Ubc9 mRNA levels experienced poorer overall survival than those displaying lower levels (Fig. 6e, left and middle panel, *P* = 0.0007 and 0.0016, respectively). Analysis of the combined effect of Slug and Ubc9 on patient prognosis revealed that patients displaying both lower Slug and Ubc9 mRNA levels had best overall survival than those showing either higher Slug and Ubc9 level, with those showing both higher Slug and Ubc9 level had worst outcomes (Fig. 6e, right panel, *P* = 0.0002). Multivariate Cox survival regression showed that both higher Slug and Ubc9 mRNA levels (Hazard Ratio [HR] = 5.08, 95% confidence interval [CI] = 1.99–12.92; *P* = 0.001) and more advanced tumor stage were independent prognostic factors for overall survival (Table 2).

**Table 1:** Characteristics of 104 NSCLC patients in real-time quantitative RT-PCR analysis^a^

Parameter	No. of patients	Low Slug (%)	High Slug (%)	*P*	Low Ubc9 (%)	High Ubc9(%)	*P*
Number of patients	104	66 (63.5)	38 (36.5)		48 (46.2)	56 (53.8)	
Sex							
Male	77	46 (69.7)	31 (81.6)		37 (77.1)	40 (71.4)	
Female	27	20 (30.3)	7 (18.4)	0.183	11 (22.9)	16 (28.6)	0.512
Histological type							
Squamous cell carcinoma	37	19 (28.8)	18 (47.4)		19 (39.6)	18 (32.1)	
Adenocarcinoma	62	44 (66.7)	18 (47.4)		28 (58.3)	34 (60.7)	
Others	5	3 (4.6)	2 (5.3)	0.144	1 (2.1)	4 (7.1)	0.406
Tumor size^b^							
≤ 3 cm	7	5 (7.7)	2 (5.3)		2 (4.3)	5 (8.9)	
> 3 cm	96	60 (92.3)	36 (94.7)	0.637	45 (95.7)	51 (91.1)	0.348
Tumor stage^c^							
Stage I	42	32 (50.0)	10 (26.3)		22 (46.8)	20 (36.4)	
Stage II	20	11 (17.2)	9 (23.7)		8 (17.0)	12 (21.8)	
Stage III–IV	40	21 (32.8)	19 (50.0)	0.062	17 (36.2)	23 (41.8)	0.556
Slug expression							
Low	66				38 (79.2)	28 (50.0)	
High	38				10 (20.8)	28 (50.0)	0.002
Ubc9 expression							
Low	48	38 (57.6)	10 (26.3)				
High	56	28 (42.4)	28 (73.7)	0.002			

^a^*P* values were calculated using a two-sided Pearson Chi-Squared test. Slug and Ubc9 expression was designated as ‘high’ or ‘low’ using 0.6 as the cut-off point

^b^One patient’ tumor size state was missing

^c^Two patients’ tumor stage states were missing

**Table 2:** Hazard ratios for death (from any cause) among patients with NSCLC based on gene expression levels as determined via real-time quantitative RT-PCR and other parameters according to multivariate Cox regression analysis^a^

Parameter	Hazard ratio (95% CI)	*P*
High Slug and Ubc9 expression	5.08 (1.99 to 12.92)	0.001
Other expression profiles	2.20 (0.82 to 5.91)	0.118
Sex	0.54 (0.24 to 1.20)	0.130
Histological type	0.66 (0.32 to 1.38)	0.268
Tumor size	1.04 (0.28 to 3.85)	0.949
Tumor stage	2.22 (1.48 to 3.33)	0.0001

^a^A stepwise method was used to select the optimal multivariate Cox proportional hazard regression model. Slug and Ubc9 expression was designated as ‘high’ or ‘low’ using 0.6 as the cut-off value (low Slug and low Ubc9 as the references), and the results were adjusted according to sex (female as the reference vs. male), histological type (squamous cell carcinoma as the reference vs. adenocarcinoma), tumor size (≤ 3 cm as the reference vs. > 3 cm), and tumor stage. *P* values (two-sided) were calculated using a Pearson Chi-square test. Abbreviations: *CI* confidence interval

Then, the studies were also re-confirmed by examining the protein expression levels of Slug and Ubc9 in tumor specimens from another NSCLC cohort (*n* = 85, Table 3). As Fig. 6f shown, patients presented with both higher Slug and Ubc9 expression levels experienced similarly poorer overall survival than those displaying both lower levels (*P* = 0.0177). Multivariate Cox proportional hazard regression also was confirmatory (Table 4, HR = 5.08, 95% CI = 1.55–16.67; *P* = 0.007).

**Table 3:** Characteristics of 85 NSCLC patients in Immunohistochemistry staining analysis^a^

Parameter	No. of patients	Low Slug (%)	High Slug (%)	*P*	Low Ubc9 (%)	High Ubc9 (%)	*P*
Number of patients	85	42 (49.4)	43 (50.6)		55 (64.7)	30 (35.3)	
Age (mean±SD)	63.4±9.9	62.6±10.0	64.2±9.8	0.462^2^	62.2±10.3	65.7±8.9	0.114^b^
Sex							
Male	53	30 (47.6)	23 (39.0)		31 (56.4)	13 (43.3)	
Female	69	33 (52.4)	36 (61.0)	0.336	24 (43.6)	17 (56.7)	0.251
Histological type							
Squamous cell carcinoma	11	5 (11.9)	6 (14.0)		11 (2.0)	0 (0.0)	
Adenocarcinoma	68	32 (76.2)	36 (83.7)		41 (74.5)	27 (90.0)	
Others	6	5 (11.9)	1 (2.3)	0.225	3 (5.5)	3 (10.0)	0.028
Tumor size							
≤3cm	33	16 (38.1)	17 (39.5)		21 (38.2)	12 (40.0)	
>3cm	52	26 (61.9)	26 (60.5)	0.892	34 (61.8)	18 (60.0)	0.869
Lymph node metastasis							
Negative	39	18 (42.9)	21 (48.8)		23 (41.8)	16 (53.3)	
Positive	46	24 (57.1)	22 (51.2)	0.580	32 (58.2)	14 (46.7)	0.309
Tumor size^c^							
Stage I	30	15 (35.7)	15 (34.9)		24 (43.6)	6 (20.0)	
Stage II	32	15 (35.7)	18 (41.9)		18 (32.7)	15 (50.0)	
Stage III–IV	19	10 (23.8)	9 (20.9)	0.871	12 (21.8)	7 (23.3)	0.100
Slug expression							
Low					30 (54.5)	12 (40.0)	
High					25 (45.5)	18 (60.0)	0.200
Ubc9 expression							
Low		30 (71.4)	25 (58.1)				
High		12 (28.6)	18 (41.9)	0.200			

^a^*P* values were calculated using a two-sided Pearson Chi-Squared test. Slug and Ubc9 expression was designated as ‘high’ or ‘low’ using “50 and 60% immunoreactivity was shown in tumor sections” as the cut-off point respectively

^b^*P* value was calculated using a Student’s *t*-test

^c^Three patients’ tumor stage states were missing

**Table 4:** Hazard ratios for death among patients with NSCLC based on protein expression levels as determined via immunohistochemistry and other parameters according to multivariate Cox regression analysis^a^

Parameter	Hazard ratio (95% CI)	*P*
High Slug and Ubc9 expression	5.08 (1.55 to 16.67)	0.007
Other expression profiles	2.15 (0.72 to 6.39)	0.168
Sex	2.45 (1.13 to 5.31)	0.023
Histological type	0.63 (0.23 to 1.70)	0.361
Tumor size	1.21 (0.96 to 1.51)	0.104
Tumor stage	0.94 (0.60 to 1.49)	0.800

^a^A stepwise method was used to select the optimal multivariate Cox proportional hazard regression model. Slug and Ubc9 expression was designated as ‘high’ or ‘low’ using “50% and 60% immunoreactivity in the tumor sections” as the respective cut-off values (low Slug and low Ubc9 as the references). The results were adjusted according to sex (female as the reference vs. male), histological type (squamous cell carcinoma as the reference vs. adenocarcinoma), tumor size (≤ 3 cm as the reference vs. > 3 cm), and tumor stage. *P* values (two-sided) were calculated using a Pearson Chi-square test. Abbreviations: CI, confidence interval

Collectively, all these data indicate the malignant role of Slug SUMOylation in lung cancer and also highlight the importance to dissect the regulation mechanism of Slug SUMOylation.

## Discussion

**Fig. 7:**
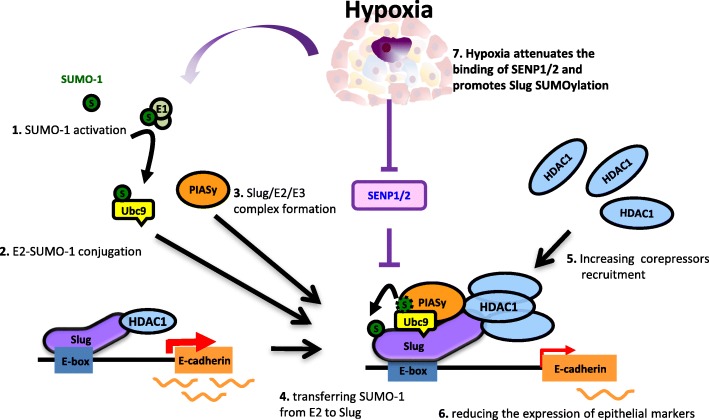
Provisional model of Hypoxia-induced SUMOylation in cancer metastasis. **1**. The SUMO-1 protein is activated by conjugation to the E1 heterodimer. **2**. SUMO-1 is subsequently transferred from an E1 to the unique E2 enzyme Ubc9. **3**. Ubc9–SUMO-1 and E3 (PIAS family) directly bind to chromatin-bound Slug. **4**. SUMO-1 covalently conjugates to the *C*-terminus of Slug. **5**. Slug SUMOylation increases the recruitment of corepressors, such as Sin3A, CtBP, HDAC1 and HDAC2, to its *N*-terminus. **6**. Then, Slug represses the expression of target genes associated with the epithelial markers such as E-cadherin, occludin, claudin-1, and integrin α3 to promote cancer metastasis. **7**. Hypoxia increases Slug SUMOylation by attenuating its binding to SENP1/2. In conclusion, SUMOylation further enhances Slug-mediated cancer cell migration and invasion and tumor metastasis

Slug participates in the regulation of cell migration, apoptosis, differentiation, and therefore can promote cancer invasion and metastasis [1, 4, 41–43]. Our study demonstrates that Slug could be SUMOylated at its *C*-terminus via direct binding to Ubc9 and PIASy on chromatin. Hypoxia could induce Slug SUMOylation through inhibiting the interaction between Slug and SENP1/2 with result in downregulating the process of deSUMOylation. Detail analyses indicated that SUMOylation could enhance the transcriptional repression activity of Slug by recruiting more corepressors (like Sin3A, CtBP, HDAC1 or HDAC2), decreasing its downstream target gene expressions (such as E-cadherin, claudin 1 and occludin) and promoting cancer cell migration, invasion, and metastasis (Fig. 7). Moreover, the expressions of Slug and Ubc9 could be used as effective diagnosis markers for Slug SUMOylation and the combined effect was associated with poor overall survival in NSCLC patients.

Previously, Slug protein had been reported could be stabilized by ARF and promote prostate tumorigenesis [44]. In that report, Xie and his colleagues found that ARF could stabilize Slug through interacting with SUMO molecule at lysine residue 192 (K192), modulate the Slug/E-cadherin signaling, and augment prostate tumorigenesis in vivo*;* the regulation mechanisms of Slug SUMOylation are different with our findings. In lung cancer, we found that in addition to the K192, the amino acids 213–268 of Slug are more critical for SUMOylation and responsible for the regulation of Slug transcriptional repression activity. In fact, we ever mutated all lysine residues of Slug (Slug22M) and individually substituted them with lysine residue to find out all the SUMOylation sites of Slug (Additional file 2: Figure S2a). The results showed that rather than K192, amino acids 213–268 contribute near 75% of Slug SUMOylation; and in which, only K258 is located on a known consensus sequence, while K239 and K248 are located on non-consensus regions of SUMO modification. In addition, we further discovered that hypoxia could enhance Slug SUMOylation through disrupting the interaction between Slug and SENP1/2, and result in lung cancer malignancy. All these suggest Slug protein could be SUMOylated under different regulation and affected cancer malignancy with different action mechanisms; while ARF overexpression, Slug was SUMOylated at K192 to accumulate Slug protein to promote prostate tumorigenesis in vivo; when hypoxia or Ubc9/PIASs overexpression, Slug was SUMOylated and enhanced transcriptional repression activity in lung cancer malignancy. We provided more detail molecular mechanism to state the regulation and its critical role of Slug SUMOylation in cancer.

Typically, PTMs are sophisticated and reversible; which confer the functional abilities on proteins at the appropriate time and place [45]. Emerging evidences suggest that Snail and Slug could be post-translationally regulated by phosphorylation, ubiquitination, SUMOylation and acetylation [44, 46–49]; and the latter three are all occurred on lysine residue. Moreover, literature also indicates that the SUMOylation and ubiquitination pathways could intersect and communicate with each other [50]. These let us curious about whether exist any relationship between ubiquitination, acetylation and Slug SUMOyaltion. To answer this question, we further examined and dissected the responsible regions of Slug ubiquitination and acetylation. The data showed that Slug could be ubiquitinated at K116 (Additional file 14: Figure S14a) that different from the sites for SUMOylation; and with nearly non-acetylation in the steady state (Additional file 14: Figure S14b). Previously, Vernon et al. reported that the Slug *N*-terminus is responsible for its ubiquitin-mediated proteasomal degradation [47]; but we found that Slug is ubiquitinated on K116. These imply that ubiquitination and SUMOylation may occur in different Slug regions. In addition, the data of none appreciable acetylation existed in Slug protein was different with the finding that acetylation of Snail proteins could determine its role as an activator or repressor in cells [49]. Moreover, we also observed that SUMOylation predominantly occurred on Slug rather than on Snail (Additional file 15: Figure S15). Whether the processes of these PTMs communicate with each other should be further clarified.

As we know, SUMOylation participates in functional regulation of many proteins; impaired regulation may lead human diseases like cancer, neurodegenerative disorder, cardiac disease, and ocular pathology [51–53]. Till now, several studies mentioned that SUMO E1, E2, and E3 enzymes such as Ubc9 and PIAS3 are upregulated in many cancer types [22, 54–57]. Though disturbing SUMOylation could interfere several protein functions that relevant to cancer progression [58, 59], the detail mechanism how SUMO promotes tumorigenesis remains unclear. Here, we provide a potential mechanism by which SUMO-1 could participate in cancer metastasis through binding to Slug. The investigation showed that Slug SUMOylation might increase corepressor, HDAC1, recruitment, inhibit downstream target genes expressions, and have end result in promoting cancer malignancy. Similar findings were observed in SUMOylated Elk-1 and p300 that SUMOylation might control transcriptional repression by recruitment of HDAC2 and HDAC6; and also mentioned in previous reports [60–63].

Hypoxia, a common characteristic in most malignant tumors, stimulates a complex cancer-related gene expression and is also involved in SUMO signaling [37, 64–68]. In this study, we found that hypoxia could induce Slug SUMOylation by repressing the interaction between Slug and SENP1/2, though the detailed mechanism should be further clarified. While Slug can be phosphorylated, ubiquitinated and SUMOylated in cells, we observed no significant difference in Slug ubiquitination between normoxia and hypoxia status (Fig. 5b). Our data also indicated that both serine and threonine phosphorylations of Slug were lower under hypoxia, suggesting Slug protein could be regulated by phosphorylation or SUMOylation under hypoxia. Moreover, the Slug interactome contained several subunits of protein phosphatase 2 (PP2A), such as PPP2CB, PPP2R1A, PPP2R2A and PPP2R2D [37]; as such, we further discovered that the PP2 inhibitor okadaic acid exposure could reverse the hypoxia-induced increasing Slug SUMOylation (Additional file 16: Figure S16). All these findings implied that Slug phosphorylation and SUMOylation were related to PP2 activity under hypoxia analogue to prior report [69].

## Conclusions

In sum, our study provided new insight into the modulation of the EMT regulator Slug via SUMOylation and disclosed novel mechanisms by which SUMOylated Slug promotes cancer invasion and metastasis under hypoxia. The identification of these findings may have clinical implications in targeting lung cancer treatment.

## Additional files

Additional file 1: **Figure S1.** Slug is primarily modified by SUMO-1. Slug is primarily SUMOylated by SUMO-1 in vivo. Lysates of HEK293T cells were cotransfected with plasmids encoding 3xFlag-tagged Slug and different GFP-tagged isoforms of SUMO. The lysates were collected and used for immunoprecipitation with an anti-Flag antibody. β-actin was used as the internal control. The asterisk and arrowhead indicate Slug modified and not modified by SUMO-1, respectively. (PDF 99 kb)

Additional file 2: **Figure S2.** The activities of Slug mutants. (**a**) Mutation of individual lysine affects the SUMOylated level of Slug. HEK293T cells were cotransfected with plasmids encoding different 3xFlag-tagged Slug mutants and GFP-tagged SUMO-1. The lysates were used to examine the SUMOylation levels by immunoblotting with anti-Flag antibodies. (**b**) Different levels of SUMOylation between Slug mutants. HEK293T cells were transfected with expression vectors encoding GFP-tagged SUMO-1 and different 3xFlag-tagged Slug mutants (22 M, all lysines were replaced with arginines; 5 M: lysines at 239, 240, 244, 248, and 258 were replaced with arginines; 6 M: lysines at 188, 239, 240, 244, 248, and 258 were replaced with arginines). These lysates were also examined by immunoblotting with anti-Flag antibodies. The asterisk and arrowhead indicate Slug modified and not modified by SUMO-1, respectively. (**c**) The transcriptional repression activity of wild-type and mutant Slug proteins. HEK293T cells were cotransfected with the SBS–Gal4–luciferase reporter and Gal4–VP16 activator expression plasmids together with the wild-type or mutant Slug expression plasmid (8 M: lysines at 135, 145, 188, 239, 240, 244, 248, and 258 were replaced with arginines), and the luciferase assay was performed to determine the transcriptional repression activity of Slug. Immunoblotting results are presented alongside the luciferase assay results to demonstrate the expression of the Slug mutant proteins. (**d**) The DNA-binding activity of wild-type and mutant Slug proteins. The wild-type and mutant Slug proteins used in the EMSA were produced using an in vitro transcription/translation system. The protein expression levels were evaluated by immunoblotting with anti-Slug antibodies (top panel). Phosphor image analysis of the EMSA gel showing ^32^P-labeled E-box oligonucleotides incubated with in vitro-translated proteins (4 μl) or with Slug antibodies (Ab: antibody, 0.3 μg) (bottom panel). (PDF 152 kb)

Additional file 3: **Figure S3.** The Slug protein levels reflect its SUMOylated levels. To correlate the protein expression levels with the levels of SUMOylation, we subcutaneously injected KEK293 cells overexpressing Slug/vector control or Slug/HA–Ubc9 into mice. Tumor tissues were removed at 42 days after tumor injection and then lysed with tissue protein extraction reagent contained proteinase inhibitors and NEM. Subsequently, the samples were subjected to immunoprecipitation with an anti-Slug antibody prior to immunoblotting with the indicated antibodies. β-actin was used as the internal control. The asterisk and arrowhead indicate Slug modified and not modified by ubiquitin, respectively. (PDF 26 kb)

Additional file 4: **Figure S4.** Direct interaction of Slug with PIAS family members. A pull-down assay was used to determine the physical interaction between Slug and PIAS family members. Recombinant GST and GST–Slug proteins were produced from bacteria, and the translated products of HA-tagged PIAS family member genes were obtained using an in vitro transcription/translation system. The production of these proteins was demonstrated by immunoblotting using anti-GST and anti-HA antibodies, respectively. GST–Slug was used in the pull-down assay for in vitro interaction with HA-tagged PIAS family members. The GST protein alone was used as a negative control. (PDF 24 kb)

Additional file 5: **Figure S5.** Structure of the Slug/PIASy/Ubc9/SUMO-1 complex. (**a**) Schematic showing the regions of Slug that interact with PIASy, Ubc9, and SUMO. Slug is 268 amino acids in length and contains a SNAG repression domain at its N-terminus and five zinc finger (ZnF) domains at its C-terminus. ND means no detection. (**b**) A 3D structure of Slug/PIASy/Ubc9/SUMO-1 complex was generated using prediction software (orange, Slug; purple, PIASy; green, Ubc9; gray, SUMO-1). A rotated view of this complex is shown in the lower panel. (PDF 127 kb)

Additional file 6: **Figure S6.** Characterization of Slug and Slug5M protein. (**a**) The DNA-binding ability of Slug is not altered by the inserted mutations. Equal amounts of in vitro-translated Slug and Slug5M were used in the EMSAs (left panel). Slug and Slug5M bound to the E-box C probes in a dose-dependent manner (+: 0.1 μl; ++: 0.3 μl; +++: 1 μl) (right panel). Anti-Slug antibodies were used to confirm that the shifted bands were formed specifically by Slug and Slug5M. (**b**) The protein stability of Slug is not altered by the inserted mutations. Protein stability was not significantly different between the wild-type and mutant forms of Slug. Slug- and Slug5M-overexpressing HEK293 cells were treated with cycloheximide (CHX) to prevent further protein synthesis for the indicated periods. The expression of Slug was analyzed by immunoblotting. β-actin was used as the internal control. Relative densitometry results are plotted in the bottom panel. (PDF 68 kb)

Additional file 7: **Figure S7.** Slug recruits corepressors more abundantly than Slug5M. The nuclear fractions of Slug- and Slug5M-overexpressing HEK293 cells were obtained by adding hypotonic buffer to the cells. Subsequently, the samples were subjected to immunoprecipitation using an anti-Slug antibody. The accompanying precipitates were analyzed by immunoblotting using the indicated antibodies (left panel). Lamin B was used as a nuclear marker. The relative densitometry results (the results for Slug were normalized to one) were calculated using two programs (ImageJ and GelPro3.1), and the average values are plotted in the right panel. (PDF 61 kb)

Additional file 8: **Figure S8.** HDAC1 stronger associated with the E-cadherin promoter in Hop62 cells overexpressing Ubc9 compare with the vector control cells. The normal mouse IgG and HDAC1 antibodies were used to pull down protein-DNA complexes in Hop62 cells with or without Ubc9 overexpression; and the E-cadherin promoter level in the samples was determined by PCR using a gene-specific primer set. Input, an aliquot of each sample was prepared and used as a template for PCR to examine the level of the E-cadherin promoter before immunoprecipitation (IP). (PDF 25 kb)

Additional file 9: **Figure S9.** SUMOylation affects the expression of Slug-regulated downstream targets. (**a**) CL1–5 cells were induced to express the wild-type and mutant Slug, respectively, using a lentiviral system. The mRNA expression of the indicated genes was determined via RT-PCR. Gβ-like was used as the internal control. (**b**) The protein expression of the Slug downstream target, E-cadherin, in HEK293 (left) and CL1–2 (right) over-expressing Slug wild-type or Slug5M cells. The results were analyzed by immunoblotting with the indicated antibodies. β-actin was used as the internal control. (PDF 174 kb)

Additional file 10: **Figure S10.** Hypoxia slightly increased the interactions of Slug with Ubc9 and PIASy. (**a** and **b**) HEK293T cells were transiently transfected with the indicated plasmids and then exposed to hypoxia (1% O_2_ for 4 h). Afterwards, the cells were subjected to immunoprecipitation with an anti-Flag antibody prior to immunoblotting with the indicated antibodies. The expression levels of HIF1α were used to confirm that the cells were exposed in hypoxia. β-actin was used as the internal control. **c** Slug SUMOylation was decreased in cells overexpressing SENP1/2. HEK293T cells were cotransfected with 3xFlag–Slug, GFP–SUMO-1 and the wild-type or mutant form of HA–SENP1/2 (C/S). Slug SUMOylation was studied by immunoblotting. The asterisk and arrowhead indicate Slug modified and not modified with SUMO-1, respectively. (PDF 72 kb)

Additional file 11: **Figure S11.** Effects of Slug and Slug5M overexpression on cultured cell proliferation. HEK293 cells were driven to express wild-type or mutant Slug using a lentiviral system. Cells were counted at the indicated time points after plating. No significant difference in the cell proliferation rate was found between the different cell lines based on one-way ANOVA. Error bars indicate mean values ± SEM. (PDF 69 kb)

Additional file 12 **Figure S12.** Slug SUMOylation promotes cell migration. CL1–2 (**a**) and Hop62 (**b**) cells infected with lentivirus encoded wild-type and mutant form of Slug were analyzed the cell migratory ability in wound healing assay. Phase contrast images were captured at the beginning (0 h) and after 12 or 15 h using standard culture inserts. The migratory ability of the cells is presented in the right panel, and the control group was normalized to 100%. *P* values were calculated and compared with vector only control by Student’s *t*-test. (PDF 177 kb)

Additional file 13: **Figure S13.** Slug SUMOylation promotes cell invasion. CL1–2 (**a**) and Hop62 (**b**) cells infected with lentivirus encoded wild-type and mutant form of Slug were analyzed the cell invasive ability in modified Boyden chamber invasion assay. The invading cells were stained by Giemsa staining (left) and were quantified (right; *n* = 3). The invasive ability of the cells is presented in the right panel, and the control group was normalized to 100%. P values were calculated and compared with vector only control by Student’s *t*-test. (PDF 101 kb)

Additional file 14: **Figure S14.** The levels of ubiquitinated and acetylated Slug. (**a**) To identify the ubiquitination sites of Slug, HEK293T cells were cotransfected with plasmids encoding different 3xFlag-tagged Slug mutants and Myc-tagged ubiquitin and were then treated with the proteasome inhibitor MG132 (10 μM for 6 h). Immunoprecipitation of the Slug protein using anti-Flag antibodies was analyzed by immunoblotting with anti-Flag antibodies. The asterisk and arrowhead indicate Slug modified and not modified by ubiquitin, respectively. (**b**) The acetylation of Slug. Different 3xFlag-tagged Slug mutants were transfected into HEK293T cells in the presence or absence of the sirtuin deacetylase inhibitor NAM and the HDAC inhibitor TSA. Acetylation of Slug was evaluated via immunoprecipitation and western blot analysis. Asterisk indicated acetylated Slug. (PDF 113 kb)

Additional file 15: **Figure S15.** The Slug protein was preferentially SUMOylated. The SUMOylated levels of the Snail and Slug proteins in the steady state. HEK293T cells were cotransfected with plasmids encoding Flag-tagged Snail or Slug and GFP-tagged SUMO-1. Then, the cells were used for immunoprecipitation with an anti-Flag antibody followed by immunoblotting with the indicated antibodies. β-actin was used as the internal control. The asterisk and arrowhead indicate SUMOylated Slug and non-modified Snail and Slug, respectively. (PDF 37 kb)

Additional file 16: **Figure S16.** The hypoxia-induced enhancement of Slug SUMOylation was associated with PP2A activity. HEK293T cells were transiently transfected with 3xFlag-tagged Slug and GFP-tagged SUMO-1 and then treated with a 1-μM dose of the PP2A inhibitor okadaic acid for 1 h. After washing out the inhibitor, the cells were exposed to hypoxia (1% O2 for 4 h). The lysates were analyzed by immunoblotting with the indicated antibodies. The expression levels of HIF1α were used to confirm that the cells were exposed to hypoxia. β-actin was used as the internal control. The asterisk and arrowhead indicate Slug modified and not modified by SUMO-1, respectively. (PDF 37 kb)

## Abbreviations

EMT: Epithelial-mesenchymal transition
HDAC1: Histone deacetylase 1
HIF1α: Hypoxia-inducible factor 1-alpha
NSCLC: Non-small-cell lung cancer
PIAS: Protein inhibitor of activated STAT
SENP: Sentrin-specific protease
SUMO: Small ubiquitin-like modifier
Ubc9: SUMO-conjugating enzyme 9
