# Supplementary figures and images for: Hypoxia-induced Slug SUMOylation enhances lung cancer metastasis
Source: J Exp Clin Cancer Res. 2019 Jan 6;38:5. doi: 10.1186/s13046-018-0996-8 (PMC6322271; doi:10.1186/s13046-018-0996-8)

Hung *et al.* Figure S1

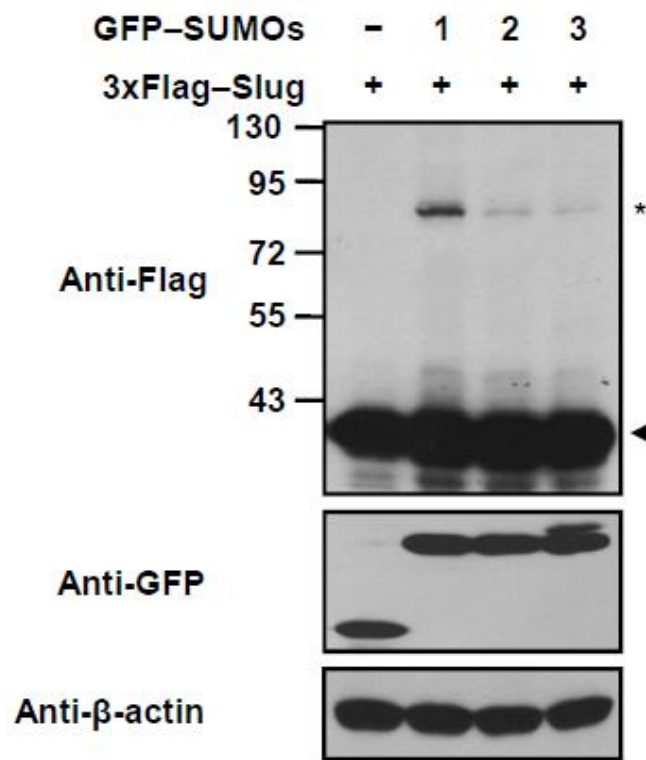

Supplement: Supplementary file 1 — Figure S1. Slug is primarily modified by SUMO-1. Slug is primarily SUMOylated by SUMO-1 in vivo. Lysates of HEK293T cells were cotransfected with plasmids encoding 3xFlag-tagged Slug and different GFP-tagged isoforms of SUMO. The lysates were collected and used for immunoprecipitation with an anti-Flag antibody. β-actin was used as the internal control. The asterisk and arrowhead indicate Slug modified and not modified by SUMO-1, respectively. (PDF 99 kb) [file 13046_2018_996_MOESM1_ESM.pdf]

Hung *et al.* Figure S2

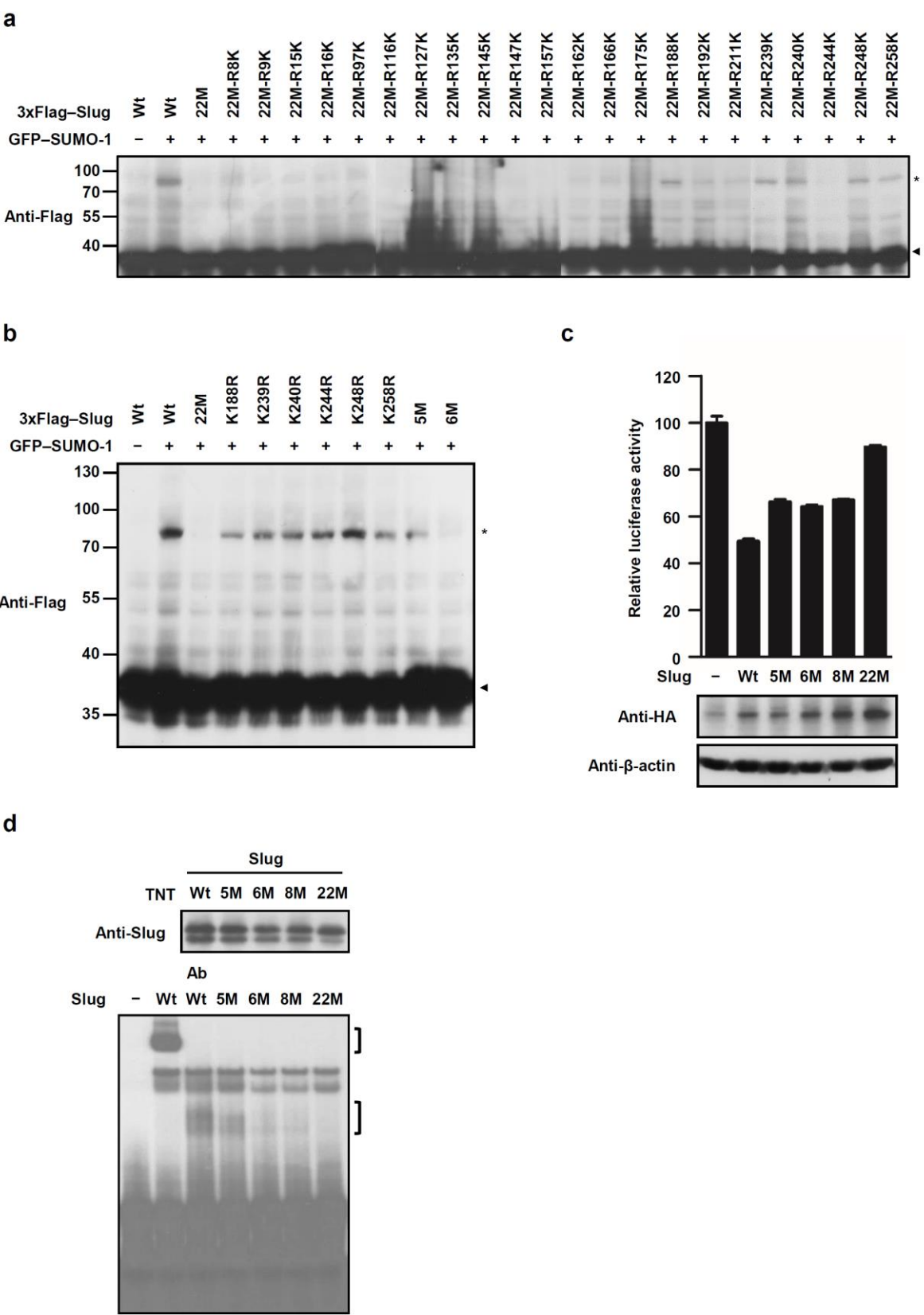

Supplement: Supplementary file 2 — Figure S2. The activities of Slug mutants. (a) Mutation of individual lysine affects the SUMOylated level of Slug. HEK293T cells were cotransfected with plasmids encoding different 3xFlag-tagged Slug mutants and GFP-tagged SUMO-1. The lysates were used to examine the SUMOylation levels by immunoblotting with anti-Flag antibodies. (b) Different levels of SUMOylation between Slug mutants. HEK293T cells were transfected with expression vectors encoding GFP-tagged SUMO-1 and different 3xFlag-tagged Slug mutants (22 M, all lysines were replaced with arginines; 5 M: lysines at 239, 240, 244, 248, and 258 were replaced with arginines; 6 M: lysines at 188, 239, 240, 244, 248, and 258 were replaced with arginines). These lysates were also examined by immunoblotting with anti-Flag antibodies. The asterisk and arrowhead indicate Slug modified and not modified by SUMO-1, respectively. (c) The transcriptional repression activity of wild-type and mutant Slug proteins. HEK293T cells were cotransfected with the SBS–Gal4–luciferase reporter and Gal4–VP16 activator expression plasmids together with the wild-type or mutant Slug expression plasmid (8 M: lysines at 135, 145, 188, 239, 240, 244, 248, and 258 were replaced with arginines), and the luciferase assay was performed to determine the transcriptional repression activity of Slug. Immunoblotting results are presented alongside the luciferase assay results to demonstrate the expression of the Slug mutant proteins. (d) The DNA-binding activity of wild-type and mutant Slug proteins. The wild-type and mutant Slug proteins used in the EMSA were produced using an in vitro transcription/translation system. The protein expression levels were evaluated by immunoblotting with anti-Slug antibodies (top panel). Phosphor image analysis of the EMSA gel showing 32P-labeled E-box oligonucleotides incubated with in vitro-translated proteins (4 μl) or with Slug antibodies (Ab: antibody, 0.3 μg) (bottom panel). (PDF 152 kb) [file 13046_2018_996_MOESM2_ESM.pdf]

Hung *et al.* Figure S3

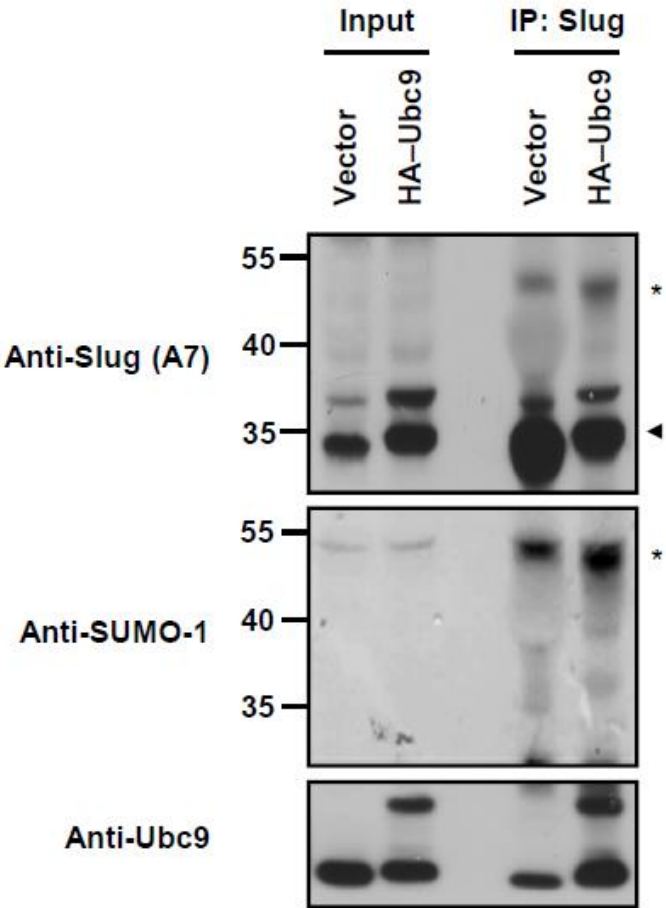

Supplement: Supplementary file 3 — Figure S3. The Slug protein levels reflect its SUMOylated levels. To correlate the protein expression levels with the levels of SUMOylation, we subcutaneously injected KEK293 cells overexpressing Slug/vector control or Slug/HA–Ubc9 into mice. Tumor tissues were removed at 42 days after tumor injection and then lysed with tissue protein extraction reagent contained proteinase inhibitors and NEM. Subsequently, the samples were subjected to immunoprecipitation with an anti-Slug antibody prior to immunoblotting with the indicated antibodies. β-actin was used as the internal control. The asterisk and arrowhead indicate Slug modified and not modified by ubiquitin, respectively. (PDF 26 kb) [file 13046_2018_996_MOESM3_ESM.pdf]

Hung *et al.* Figure S4

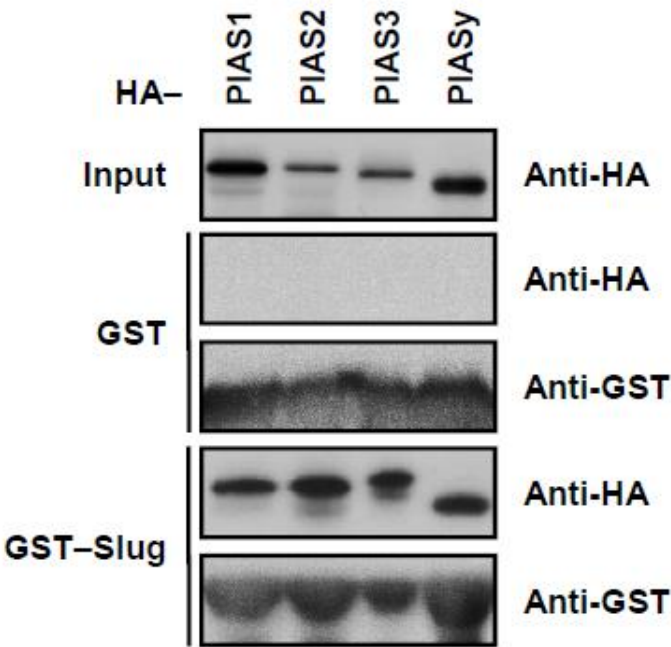

Supplement: Supplementary file 4 — Figure S4. Direct interaction of Slug with PIAS family members. A pull-down assay was used to determine the physical interaction between Slug and PIAS family members. Recombinant GST and GST–Slug proteins were produced from bacteria, and the translated products of HA-tagged PIAS family member genes were obtained using an in vitro transcription/translation system. The production of these proteins was demonstrated by immunoblotting using anti-GST and anti-HA antibodies, respectively. GST–Slug was used in the pull-down assay for in vitro interaction with HA-tagged PIAS family members. The GST protein alone was used as a negative control. (PDF 24 kb) [file 13046_2018_996_MOESM4_ESM.pdf]

Hung *et al.* Figure S5

a

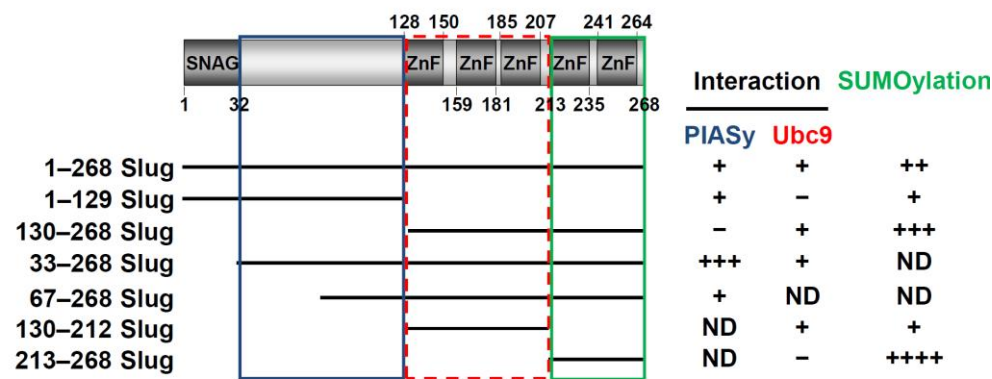

b

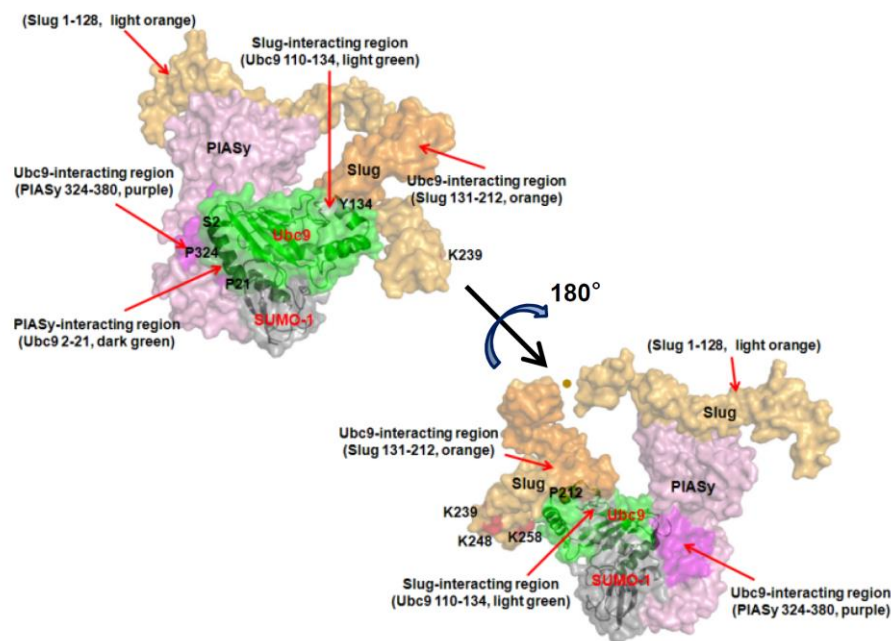

Supplement: Supplementary file 5 — Figure S5. Structure of the Slug/PIASy/Ubc9/SUMO-1 complex. (a) Schematic showing the regions of Slug that interact with PIASy, Ubc9, and SUMO. Slug is 268 amino acids in length and contains a SNAG repression domain at its N-terminus and five zinc finger (ZnF) domains at its C-terminus. ND means no detection. (b) A 3D structure of Slug/PIASy/Ubc9/SUMO-1 complex was generated using prediction software (orange, Slug; purple, PIASy; green, Ubc9; gray, SUMO-1). A rotated view of this complex is shown in the lower panel. (PDF 127 kb) [file 13046_2018_996_MOESM5_ESM.pdf]

Hung *et al.* Figure S6

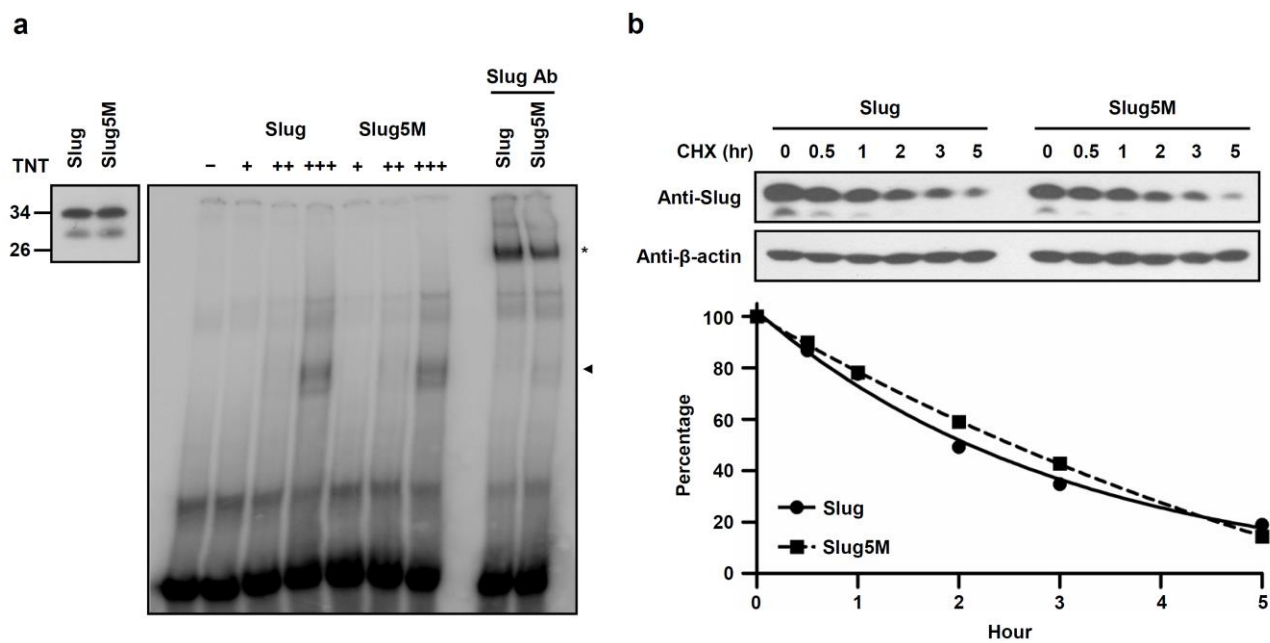

Supplement: Supplementary file 6 — Figure S6. Characterization of Slug and Slug5M protein. (a) The DNA-binding ability of Slug is not altered by the inserted mutations. Equal amounts of in vitro-translated Slug and Slug5M were used in the EMSAs (left panel). Slug and Slug5M bound to the E-box C probes in a dose-dependent manner (+: 0.1 μl; ++: 0.3 μl; +++: 1 μl) (right panel). Anti-Slug antibodies were used to confirm that the shifted bands were formed specifically by Slug and Slug5M. (b) The protein stability of Slug is not altered by the inserted mutations. Protein stability was not significantly different between the wild-type and mutant forms of Slug. Slug- and Slug5M-overexpressing HEK293 cells were treated with cycloheximide (CHX) to prevent further protein synthesis for the indicated periods. The expression of Slug was analyzed by immunoblotting. β-actin was used as the internal control. Relative densitometry results are plotted in the bottom panel. (PDF 68 kb) [file 13046_2018_996_MOESM6_ESM.pdf]

Hung *et al.* Figure S7

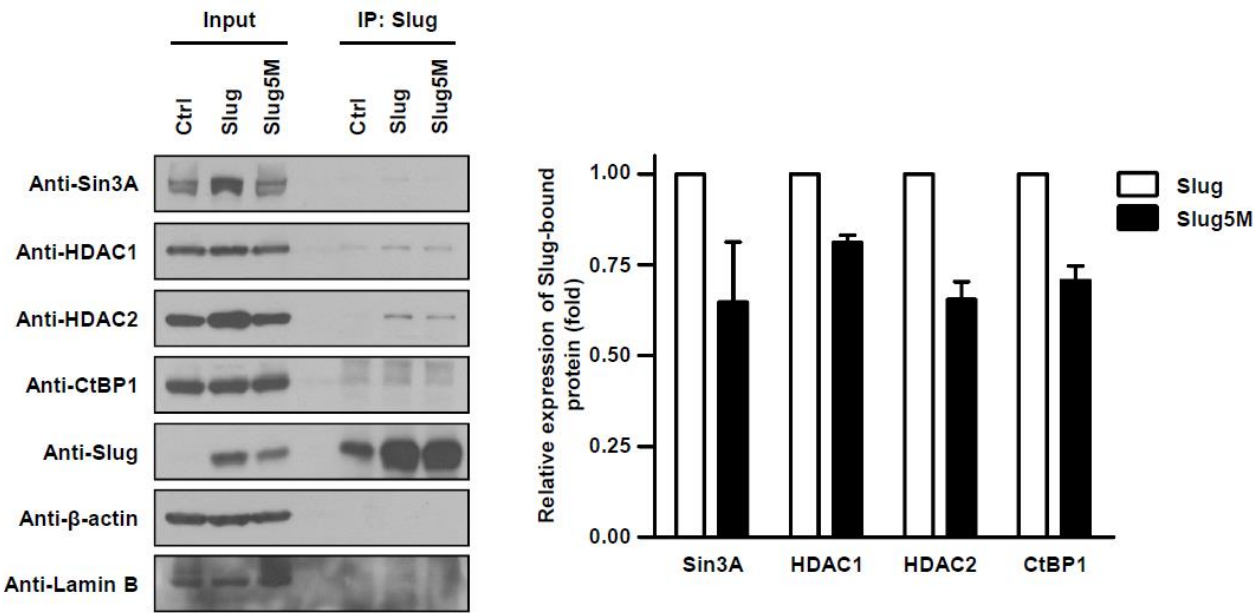

Supplement: Supplementary file 7 — Figure S7. Slug recruits corepressors more abundantly than Slug5M. The nuclear fractions of Slug- and Slug5M-overexpressing HEK293 cells were obtained by adding hypotonic buffer to the cells. Subsequently, the samples were subjected to immunoprecipitation using an anti-Slug antibody. The accompanying precipitates were analyzed by immunoblotting using the indicated antibodies (left panel). Lamin B was used as a nuclear marker. The relative densitometry results (the results for Slug were normalized to one) were calculated using two programs (ImageJ and GelPro3.1), and the average values are plotted in the right panel. (PDF 61 kb) [file 13046_2018_996_MOESM7_ESM.pdf]

Hung *et al.* Figure S8

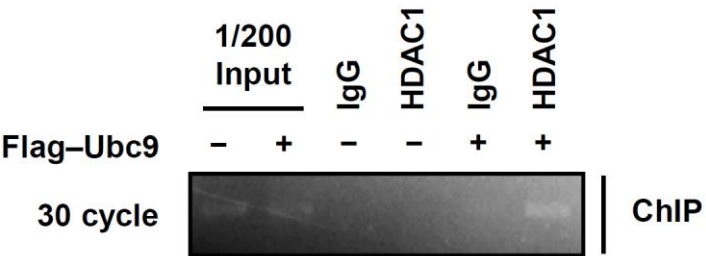

Supplement: Supplementary file 8 — Figure S8. HDAC1 stronger associated with the E-cadherin promoter in Hop62 cells overexpressing Ubc9 compare with the vector control cells. The normal mouse IgG and HDAC1 antibodies were used to pull down protein-DNA complexes in Hop62 cells with or without Ubc9 overexpression; and the E-cadherin promoter level in the samples was determined by PCR using a gene-specific primer set. Input, an aliquot of each sample was prepared and used as a template for PCR to examine the level of the E-cadherin promoter before immunoprecipitation (IP). (PDF 25 kb) [file 13046_2018_996_MOESM8_ESM.pdf]

**a**

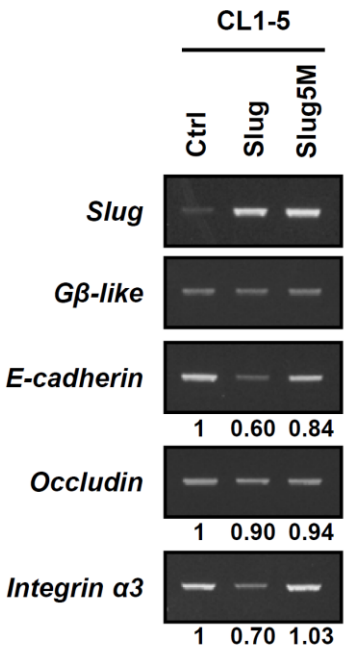

**b**

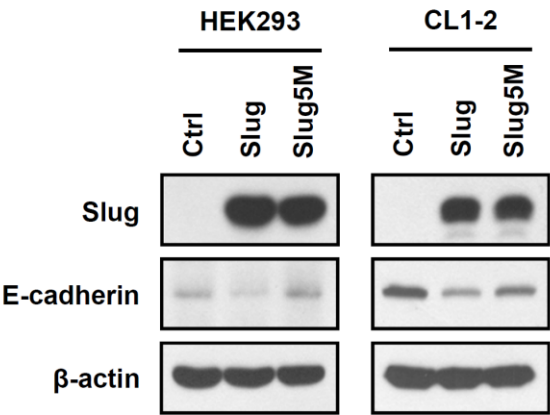

Supplement: Supplementary file 9 — Figure S9. SUMOylation affects the expression of Slug-regulated downstream targets. (a) CL1–5 cells were induced to express the wild-type and mutant Slug, respectively, using a lentiviral system. The mRNA expression of the indicated genes was determined via RT-PCR. Gβ-like was used as the internal control. (b) The protein expression of the Slug downstream target, E-cadherin, in HEK293 (left) and CL1–2 (right) over-expressing Slug wild-type or Slug5M cells. The results were analyzed by immunoblotting with the indicated antibodies. β-actin was used as the internal control. (PDF 174 kb) [file 13046_2018_996_MOESM9_ESM.pdf]

Hung *et al.* Figure S10

a

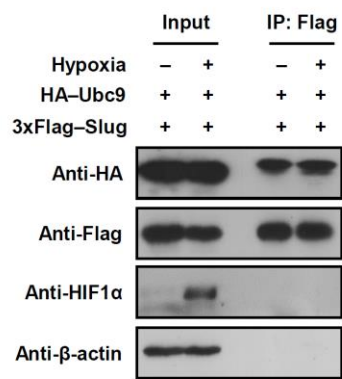

b

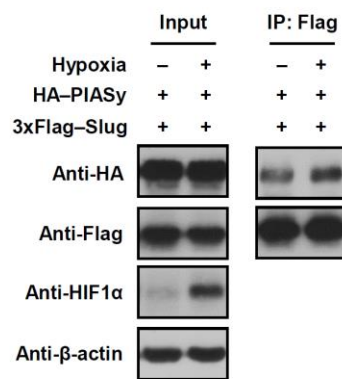

c

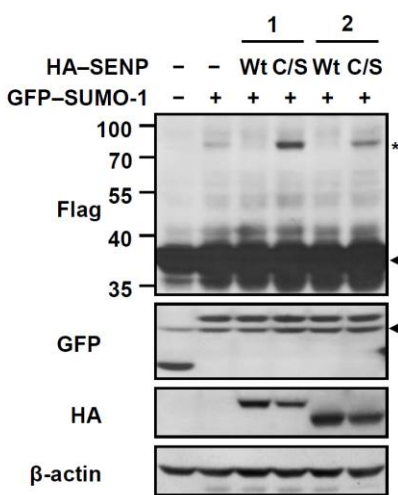

Supplement: Supplementary file 10 — Figure S10. Hypoxia slightly increased the interactions of Slug with Ubc9 and PIASy. (a and b) HEK293T cells were transiently transfected with the indicated plasmids and then exposed to hypoxia (1% O2 for 4 h). Afterwards, the cells were subjected to immunoprecipitation with an anti-Flag antibody prior to immunoblotting with the indicated antibodies. The expression levels of HIF1α were used to confirm that the cells were exposed in hypoxia. β-actin was used as the internal control. c Slug SUMOylation was decreased in cells overexpressing SENP1/2. HEK293T cells were cotransfected with 3xFlag–Slug, GFP–SUMO-1 and the wild-type or mutant form of HA–SENP1/2 (C/S). Slug SUMOylation was studied by immunoblotting. The asterisk and arrowhead indicate Slug modified and not modified with SUMO-1, respectively. (PDF 72 kb) [file 13046_2018_996_MOESM10_ESM.pdf]

Hung *et al.* Figure S11

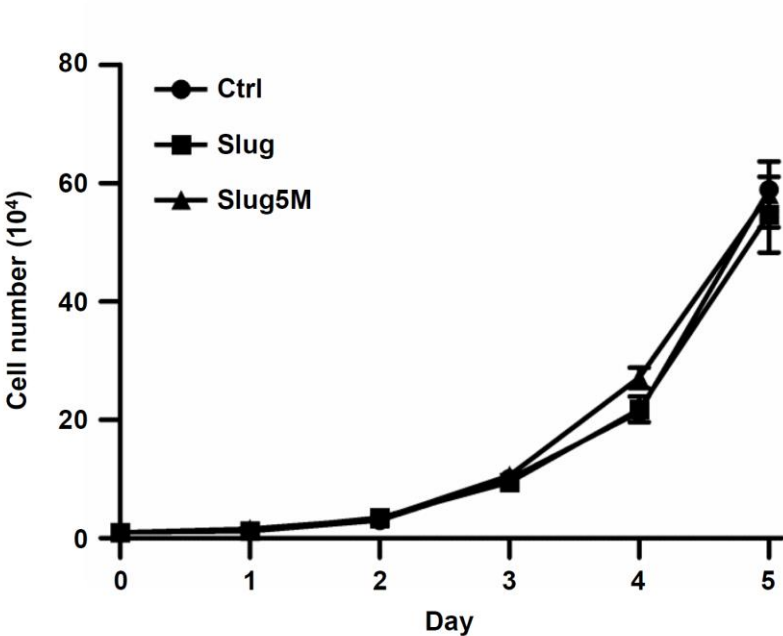

Supplement: Supplementary file 11 — Figure S11. Effects of Slug and Slug5M overexpression on cultured cell proliferation. HEK293 cells were driven to express wild-type or mutant Slug using a lentiviral system. Cells were counted at the indicated time points after plating. No significant difference in the cell proliferation rate was found between the different cell lines based on one-way ANOVA. Error bars indicate mean values ± SEM. (PDF 69 kb) [file 13046_2018_996_MOESM11_ESM.pdf]

Hung *et al.* Figure S12

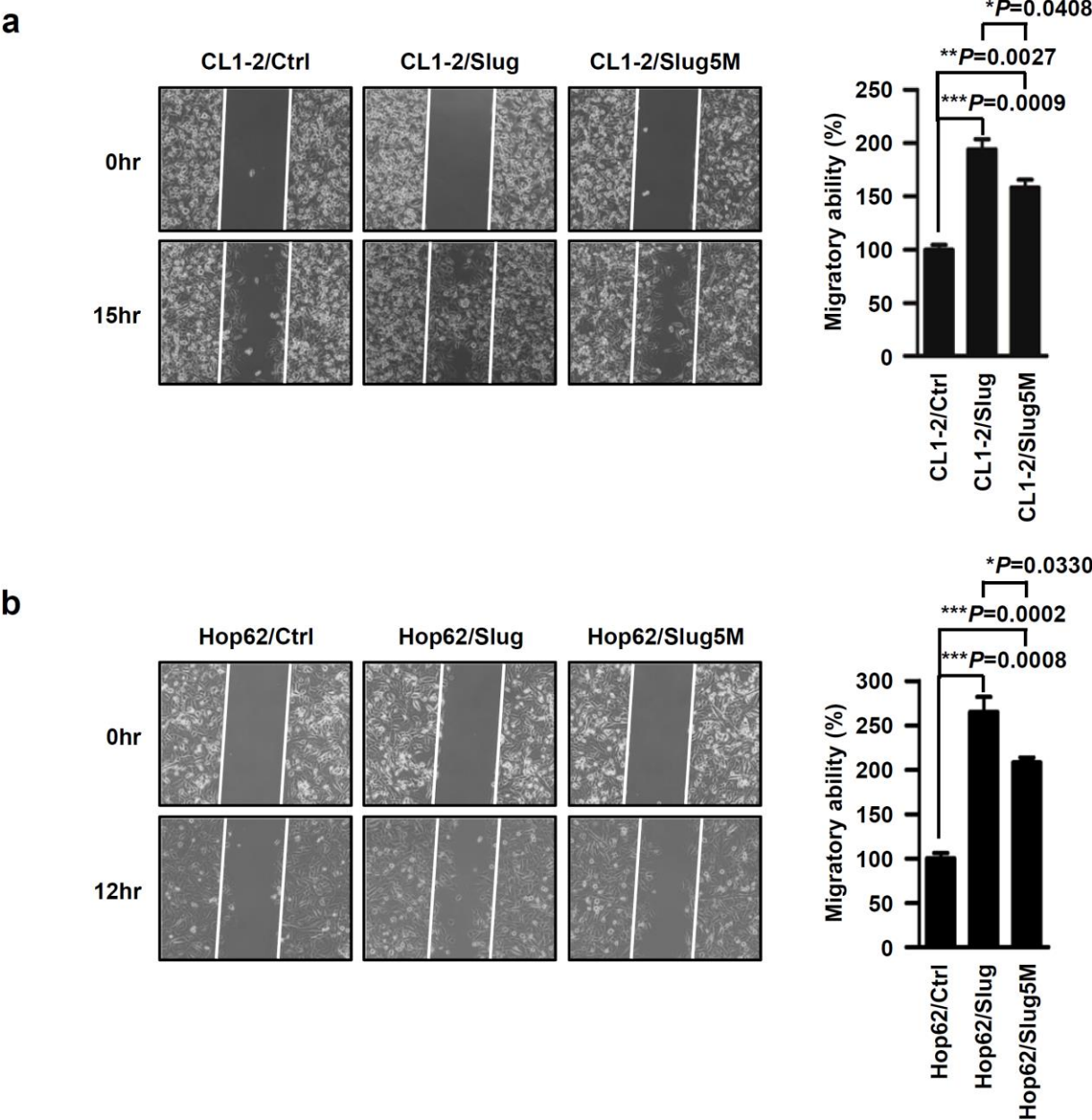

Supplement: Supplementary file 12 — Figure S12. Slug SUMOylation promotes cell migration. CL1–2 (a) and Hop62 (b) cells infected with lentivirus encoded wild-type and mutant form of Slug were analyzed the cell migratory ability in wound healing assay. Phase contrast images were captured at the beginning (0 h) and after 12 or 15 h using standard culture inserts. The migratory ability of the cells is presented in the right panel, and the control group was normalized to 100%. P values were calculated and compared with vector only control by Student’s t-test. (PDF 177 kb) [file 13046_2018_996_MOESM12_ESM.pdf]

Hung *et al.* Figure S13

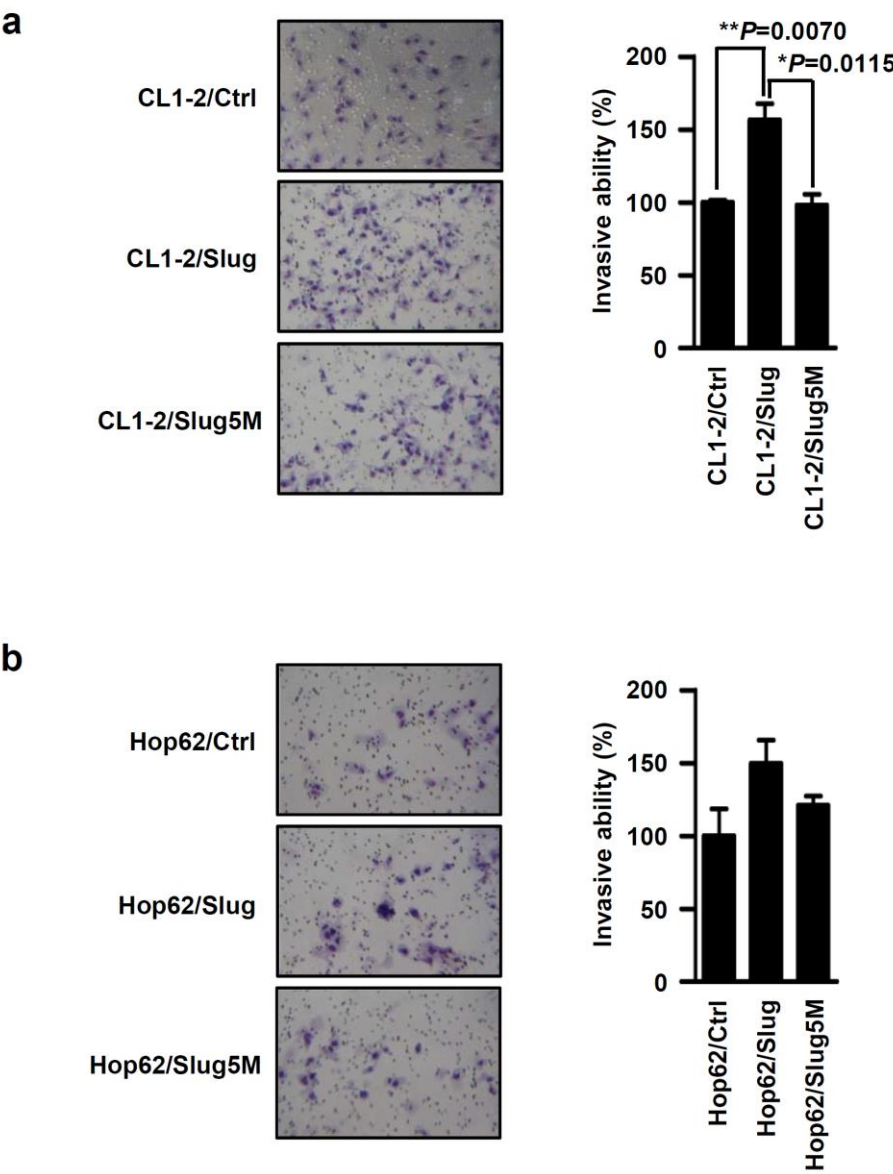

Supplement: Supplementary file 13 — Figure S13. Slug SUMOylation promotes cell invasion. CL1–2 (a) and Hop62 (b) cells infected with lentivirus encoded wild-type and mutant form of Slug were analyzed the cell invasive ability in modified Boyden chamber invasion assay. The invading cells were stained by Giemsa staining (left) and were quantified (right; n = 3). The invasive ability of the cells is presented in the right panel, and the control group was normalized to 100%. P values were calculated and compared with vector only control by Student’s t-test. (PDF 101 kb) [file 13046_2018_996_MOESM13_ESM.pdf]

Hung *et al.* Figure S14

**a**

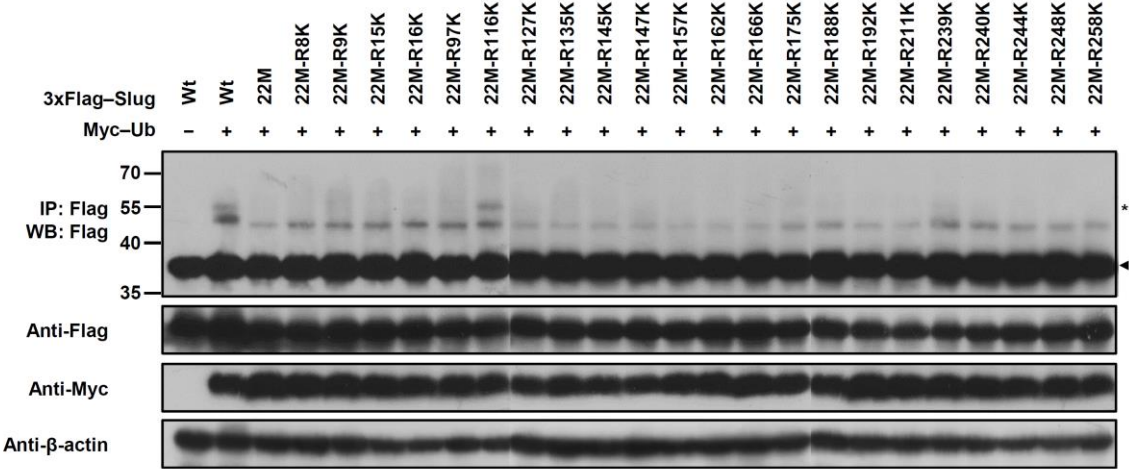

**b**

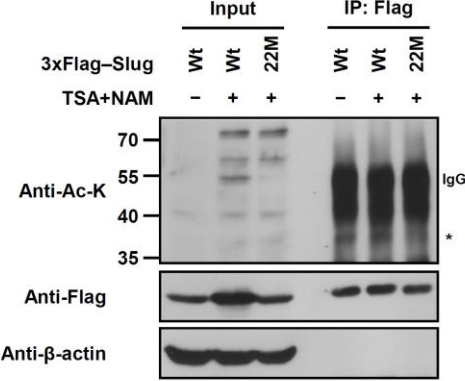

Supplement: Supplementary file 14 — Figure S14. The levels of ubiquitinated and acetylated Slug. (a) To identify the ubiquitination sites of Slug, HEK293T cells were cotransfected with plasmids encoding different 3xFlag-tagged Slug mutants and Myc-tagged ubiquitin and were then treated with the proteasome inhibitor MG132 (10 μM for 6 h). Immunoprecipitation of the Slug protein using anti-Flag antibodies was analyzed by immunoblotting with anti-Flag antibodies. The asterisk and arrowhead indicate Slug modified and not modified by ubiquitin, respectively. (b) The acetylation of Slug. Different 3xFlag-tagged Slug mutants were transfected into HEK293T cells in the presence or absence of the sirtuin deacetylase inhibitor NAM and the HDAC inhibitor TSA. Acetylation of Slug was evaluated via immunoprecipitation and western blot analysis. Asterisk indicated acetylated Slug. (PDF 113 kb) [file 13046_2018_996_MOESM14_ESM.pdf]

Hung *et al.* Figure S15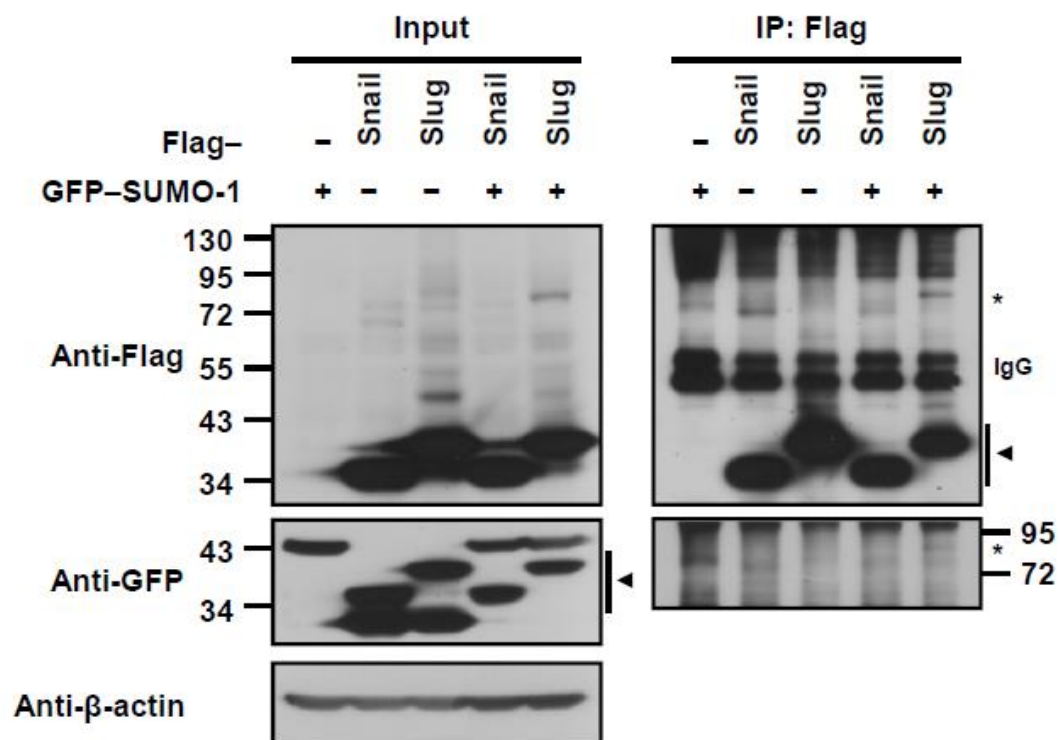

Supplement: Supplementary file 15 — Figure S15. The Slug protein was preferentially SUMOylated. The SUMOylated levels of the Snail and Slug proteins in the steady state. HEK293T cells were cotransfected with plasmids encoding Flag-tagged Snail or Slug and GFP-tagged SUMO-1. Then, the cells were used for immunoprecipitation with an anti-Flag antibody followed by immunoblotting with the indicated antibodies. β-actin was used as the internal control. The asterisk and arrowhead indicate SUMOylated Slug and non-modified Snail and Slug, respectively. (PDF 37 kb) [file 13046_2018_996_MOESM15_ESM.pdf]

Hung *et al.* Figure S16

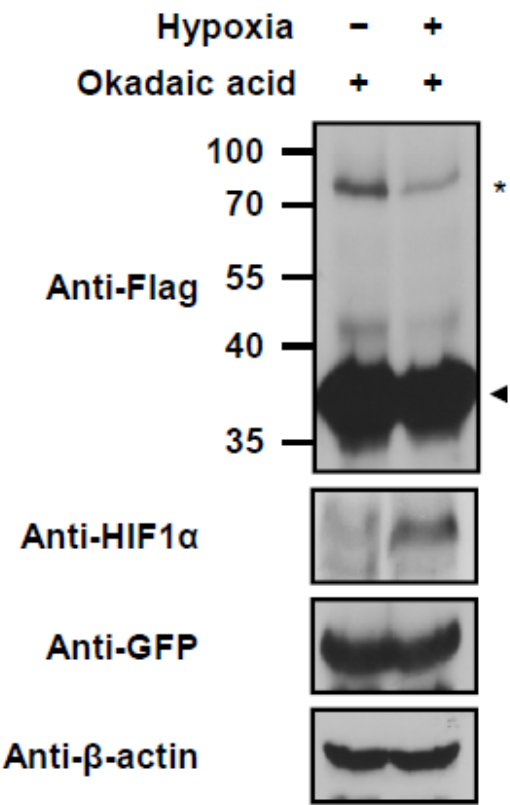

Supplement: Supplementary file 16 — Figure S16. The hypoxia-induced enhancement of Slug SUMOylation was associated with PP2A activity. HEK293T cells were transiently transfected with 3xFlag-tagged Slug and GFP-tagged SUMO-1 and then treated with a 1-μM dose of the PP2A inhibitor okadaic acid for 1 h. After washing out the inhibitor, the cells were exposed to hypoxia (1% O2 for 4 h). The lysates were analyzed by immunoblotting with the indicated antibodies. The expression levels of HIF1α were used to confirm that the cells were exposed to hypoxia. β-actin was used as the internal control. The asterisk and arrowhead indicate Slug modified and not modified by SUMO-1, respectively. (PDF 37 kb) [file 13046_2018_996_MOESM16_ESM.pdf]
